# TPI1 enhances gemcitabine resistance in bladder cancer by promoting autophagy through activating Beclin-1

**DOI:** 10.1038/s41419-025-08368-4

**Published:** 2025-12-22

**Authors:** Chenyang Wang, Kunpeng Li, Shun Wan, Siyu Chen, Shanhui Liu, Li Yang

**Affiliations:** 1 Department of Urology, Lanzhou University Second Hospital, Lanzhou, China; 2 Gansu Province Clinical Research Center for Urology, Lanzhou University Second Hospital, Lanzhou, China

**Keywords:** Bladder cancer, Mitophagy

## Abstract

The persistent issues of drug resistance and tumor recurrence remain major challenges in bladder cancer (BCa) treatment, severely impacting patient outcomes. In this study, we found that Triosephosphate isomerase 1 (TPI1) plays a crucial role in influencing gemcitabine (Gem) resistance in BCa. TPI1 is significantly upregulated in Gem-resistant BCa tissues, and the knockdown of TPI1 markedly increases Gem sensitivity and chemotherapy-induced apoptosis both in vivo and in vitro. Meanwhile, the same was validated in Gem-resistant strains. Mechanistically, transcriptome sequencing and transmission electron microscopy, among others, revealed that TPI1 promoted Gem-associated autophagy. Furthermore, mass spectrometry and co-immunoprecipitation assays demonstrated that TPI1 directly binds to the BH3 domain of Beclin-1. This interaction competitively disrupts the binding between Bcl-2 and Beclin-1, thereby relieving Bcl-2-mediated inhibition of Beclin-1. Furthermore, the interaction between TPI1 and Beclin-1 promotes the formation of PIK3C3-C1, which in turn enhances the interaction between PIK3C3-C1 and the ULK1 complex, thereby increasing the phosphorylation of Beclin-1 at Ser15. In addition, TPI1 also enhanced mitochondrial autophagy induced by Gem in BCa cells and tissues. Importantly, a transcription factor, c-Myc, that regulates TPI1 expression was also identified, and dual luciferase and Chromatin immunoprecipitation-quantitative PCR (ChIP-qPCR) analysis showed that c-Myc binds primarily to the promoter region of TPI1. Our results suggest that TPI1 plays an important role in regulating the formation of autophagic complexes, and that promoting autophagy significantly increased Gem resistance in BCa.

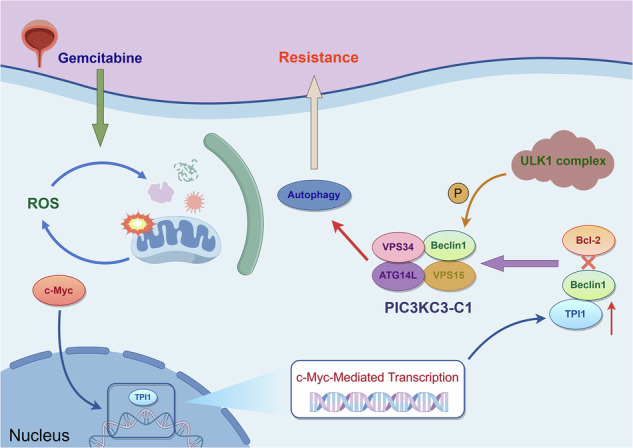

## Introduction

Bladder cancer (BCa) is a common malignant tumor of the urinary system, with its incidence and mortality rates steadily increasing in recent years [1–3]. The primary treatment for non-invasive BCa is surgical resection, while patients with advanced BCa, particularly those with metastatic disease, often receive chemotherapy to delay disease progression and improve survival [4]. In recent years, gemcitabine (Gem)-based chemotherapy has become the most widely used combination treatment for BCa, significantly improving the 5-year overall survival rate in chemotherapy-sensitive patients. However, the overall response rate to chemotherapy remains only around 20–40% [5–7]. Therefore, there is an urgent need to identify strategies to overcome chemotherapy resistance and improve clinical outcomes for BCa patients.

Autophagy is a highly conserved cellular biological process that degrades and recycles intracellular components [8]. Recent studies have revealed that autophagy is also a significant contributor to chemotherapy resistance in tumors [9, 10]. Under normal conditions, cells maintain homeostasis by clearing damaged organelles and long-lived proteins through basal autophagy. Under stress conditions, such as starvation, hypoxia, or drug stimulation, autophagy levels are significantly increased, serving as a protective mechanism to promptly remove harmful substances and provide energy to help cells survive [11–13]. Therefore, autophagy is a critical process in regulating chemotherapy resistance in tumors. It has been reported that BCa also exhibits high basal levels of autophagic activity [14, 15]. Li et al. demonstrated that starvation-induced autophagy promotes the progression of BCa cells T24 and UMUC3 through metabolic reprogramming [16]. Additionally, ENO1 enhances YAP1 expression, thereby promoting autophagy and protecting pancreatic cancer cells from Gem-induced cell death [17]. Thus, targeting autophagy in BCa appears to be a rational therapeutic approach.

In previous studies, we demonstrated that high TPI1 expression is significantly associated with poor prognosis in BCa patients and confirmed that TPI1 promotes the proliferation, invasion, and metastasis of BCa cells in both in vitro and in vivo models [18]. Additionally, we observed that TPI1 expression was significantly upregulated in tumor tissues from Gem-resistant BCa patients compared to those from Gem-sensitive patients, suggesting a potential link between TPI1 and chemotherapy resistance in BCa. However, the specific molecular mechanisms by which TPI1 modulates Gem sensitivity in BCa remain unclear. Therefore, this study aims to investigate the mechanisms through which TPI1 regulates the response of BCa cells to Gem chemotherapy, with the goal of providing new theoretical insights and therapeutic targets for reversing Gem resistance. In this study, we found that TPI1 is upregulated in chemotherapy-resistant BCa clinical specimens and influences the Gem sensitivity of BCa cells. Further experiments revealed that TPI1 enhances Gem resistance in BCa by promoting autophagy. Mechanistically, we discovered that TPI1 competitively binds to Beclin-1 with Bcl-2, thereby inhibiting the negative regulatory effect of Bcl-2 on Beclin-1. This interaction enhances autophagy in BCa cells, leading to increased Gem resistance. Additionally, these effects of TPI1 are positively transcriptionally regulated by c-Myc.

## Materials and methods

### Cell culture

The experimental study utilized two established human BCa cell lines, UMUC3 and J82, obtained from the Shanghai-based Cell Bank of the Chinese Academy of Sciences. For cell maintenance, researchers employed standard culture conditions using either RPMI 1640 or DMEM high-glucose medium, both enriched with essential supplements including 10% heat-inactivated FBS, 1% penicillin, and 1% streptomycin. Cell propagation was conducted under controlled environmental conditions, maintaining a constant temperature of 37 °C with 5% CO_2_ concentration in a humidified atmosphere.

### Cell transfection

The target cells were seeded in 6-well culture plates at a concentration of 3 × 10^5^ cells per well. Viral supernatant containing 8 µg/mL polybrene (Solarbio, Beijing) was applied to the cells, followed by a 12-h incubation period. Following transduction, the cultures were kept in standard growth medium under optimal conditions. For selection of transduced populations, puromycin (Solarbio, Beijing) was introduced at 2 µg/mL after cellular recovery. The specific sequences targeting TPI1 for interference are provided in Supplementary Table S1.

### Transcriptomic RNA sequencing

For transcriptomic RNA sequencing, total RNA was extracted from both TPI1 knockdown and control J82 cell lines following Gem treatment using TRIzol reagent (Invitrogen). Following isolation, all samples were promptly cryopreserved in liquid nitrogen. Subsequent RNA sequencing and bioinformatic analyses were performed by Majorbio Bio-Pharm Technology Co., Ltd. (Shanghai, China).

### Flow cytometry for apoptosis

Apoptosis was evaluated by flow cytometry following the Annexin V-FITC/PI Apoptosis Detection Kit protocol (Cat# AP101, Multi Sciences). Measurements were conducted on a CytoFLEX S instrument (Beckman, USA). The total apoptosis percentage was calculated by combining the proportions of early and late apoptotic cell populations.

### CCK-8 assay

For cell proliferation analysis, target cells were plated in 96-well culture plates at a concentration of 2000 cells per well. Following a 24-h incubation period under standard culture conditions (37 °C, 5% CO_2_), cells were divided into experimental groups with distinct treatment regimens, maintaining a minimum of three replicates per group. Daily assessment of cell viability was performed by adding 10 μL of CCK-8 reagent (Biosharp, Hefei) to each well and incubating for 2 h. Quantitative measurement of cellular proliferation was conducted by determining the optical density at 450 nm using a microplate reader.

### Colony formation assay

For colony formation analysis, transfected cells were plated in 6-well culture dishes at a concentration of 1000 cells per well, maintained in complete growth medium for a 10–14 day incubation period. The experimental endpoint was determined by the macroscopic observation of colony formation in the culture vessels. Post-incubation, cellular colonies were subjected to fixation using formaldehyde solution and subsequently stained with 0.1% crystal violet (Vicmed, China). Quantitative assessment was performed through photographic documentation followed by colony enumeration.

### Western blot (WB) analysis

To prepare protein samples, cells in the exponential growth phase were harvested and subjected to lysis using RIPA buffer (Solarbio, Beijing). Protein quantification was performed using the BCA Protein Assay Kit (Yamei, Shanghai) following standard protocols. Equal amounts of protein (40 μg per lane) were resolved through SDS-PAGE and subsequently transferred onto PVDF membranes. For immunodetection, membranes were initially blocked with 5% non-fat milk solution for 60 min, followed by overnight incubation with primary antibodies at 4 °C. After thorough washing, membranes were exposed to appropriate secondary antibodies for 2 h at ambient temperature, after which protein bands were visualized and quantified. The antibodies were listed in Table S2.

### Immunohistochemistry (IHC)

For immunohistochemical analysis, tissue sections underwent a sequential preparation process. Initially, xylene was employed for dewaxing, followed by rehydration through graded ethanol solutions. Antigen retrieval was performed using EDTA buffer under controlled conditions. To eliminate endogenous peroxidase activity, sections were treated with hydrogen peroxide solution. Primary antibody incubation was conducted at a dilution of 1:200, maintained at 4 °C overnight. Subsequently, sections were exposed to polyperoxidase-conjugated anti-rabbit IgG secondary antibody for 30 min at ambient temperature. Visualization was achieved through DAB chromogen development, with nuclear counterstaining accomplished using hematoxylin. The antibodies were listed in Table S2.

### Immunofluorescence

The cell samples mounted on slides underwent fixation using 4% paraformaldehyde for 30 min at room temperature. Permeabilization was then performed with 0.2% Triton X-100 solution for enhanced antibody accessibility. To prevent nonspecific binding, a blocking step was conducted with 5% bovine serum albumin for 30 min prior to overnight incubation at 4 °C with primary antibodies diluted at 1:400. Following thorough washing, fluorescent-conjugated secondary antibodies were introduced and allowed to bind for 2 h under light-protected conditions. Nuclear counterstaining was achieved through DAPI treatment, with subsequent visualization performed using a Zeiss LSM880 laser scanning confocal microscope. Complete antibody specifications are detailed in Table S2.

### Colocalization analysis

Fluorescence colocalization was assessed using a confocal microscope. To quantify the degree of colocalization between the green channel and the red channel, an intensity profile analysis was performed using ImageJ software (NIH, USA). A region of interest (ROI) was defined by a line drawn across the specific regions. The fluorescence intensity values for both the green and red channels along this line were plotted against the distance. Pearson’s correlation coefficient (Rr) was calculated to determine the correlation between the two fluorescence signals.

### Measurement of mitochondrial membrane potential and reactive oxygen species

The mitochondrial membrane potential and intracellular reactive oxygen species (ROS) levels were assessed using fluorescent probes. Following the respective treatments, cells were harvested, washed with phosphate-buffered saline, and subjected to probe staining. For mitochondrial membrane potential measurement, cells were incubated with 200 nM TMRE (Tetramethylrhodamine, ethyl ester) at 37 °C for 30 min in the dark. In contrast, for ROS detection, cells were loaded with 10 µM DCFH-DA (2′,7′-dichlorodihydrofluorescein diacetate) in serum-free medium under the same conditions. After incubation, all cells were washed thoroughly with PBS to remove excess dye. Fluorescence was immediately analyzed by fluorescence microscopy.

### Co-immunoprecipitation (Co-IP) and mass spectrometry assay

Cell lysis was performed with IP Lysis Buffer (Beaver, Suzhou) supplemented with protease inhibitor cocktail. Following centrifugation, the resulting supernatants were subjected to immunoprecipitation at 4 °C overnight using either specific antibodies or control IgG. Protein A/G magnetic beads (Beaver, Suzhou) were subsequently incubated with the antigen-antibody complexes. After extensive washing to remove nonspecific binding, the purified immunoprecipitates were processed for either WB analysis or MS detection.

### Molecular docking model

This study employed the HDOCK platform to perform protein-protein docking analysis between TPI1 (Uniprot ID: P60174) and Beclin-1 (Uniprot ID: Q14457). HDOCK integrates physically-based modeling with bioinformatics approaches to predict biomolecular interactions through optimized docking algorithms and scoring functions. The platform generates two key evaluation metrics: [1] The docking score reflects binding affinity, with more negative values indicating stronger predicted interactions; [2] The confidence score estimates binding probability, where values > 0.7 suggest high binding likelihood, scores between 0.5 and 0.7 indicate possible binding, and values < 0.5 predict low interaction probability.

### Real-Time Quantitative PCR (RT-qPCR)

Total RNA isolation from tissue specimens and cultured cells was carried out with TRIzol reagent (Invitrogen, USA). RNA concentration and purity were determined by absorbance measurements at 260 nm and 280 nm using a NanoDrop 2000 spectrophotometer (Thermo Fisher Scientific, USA), with samples demonstrating A260/A280 ratios within the optimal range of 1.8–2.0 being selected for subsequent analysis. Reverse transcription was performed on 1 µg of total RNA per sample employing M-MLV reverse transcriptase (Promega) with random hexamer primers to generate complementary DNA (cDNA). Quantitative real-time PCR amplification was conducted in triplicate reactions containing 10 µL of 2×SYBR Green master mix (Bio-Rad, USA), 2 µL cDNA template, 3 µL gene-specific primers, and 5 µL nuclease-free water. The thermal cycling protocol followed manufacturer-recommended conditions. β-actin served as the endogenous control for data normalization, with relative quantification of target gene expression determined through the comparative threshold cycle (2-ΔΔCt) method. Primer sequences used in this study are documented in Supplementary Table S3.

### Tissue specimens

BCa specimens were obtained from the Department of Urology at Lanzhou University Second Hospital, following ethical approval granted by the institution’s Research Ethics Committee. Histopathological evaluation of all BCa samples was performed by two certified pathologists to verify diagnostic characteristics. Immediately after surgical resection, tissue specimens underwent preservation through either 4% formaldehyde fixation or rapid freezing in liquid nitrogen. These processed samples were subsequently maintained at −80 °C for long-term preservation prior to experimental utilization. Inclusion criteria as well as pathological staging are shown in Table S4.

### Transmission electron microscopy (TEM)

For ultrastructural analysis, cells were initially plated in 10 cm culture dishes and allowed to proliferate until reaching 60–70% confluence. Gemcitabine (Gem) treatment was administered for a 24-h period. Post-treatment, cells were subjected to PBS washing, centrifugation, and transfer to microcentrifuge tubes. For primary fixation, 400 μL of 3% glutaraldehyde solution was added, followed by overnight incubation at 4 °C. Secondary fixation was performed using 1% osmium tetroxide (OsO_4_) at ambient temperature for 60 min. Dehydration was achieved through a graded ethanol series (30%, 50%, 70%, 90%, and 100%). The dehydrated specimens were then infiltrated and embedded in LR White resin (Santa Cruz Biotechnology, sc-215266). Ultrathin sections (60 nm) were prepared and double-stained with uranyl acetate and lead citrate. Transmission electron microscopy (TEM) was employed for ultrastructural examination, with quantitative assessment of autophagic vacuoles performed.

### Dual‑luciferase reporter assay and ChIP-qPCR

To investigate transcriptional regulation, 293 T cells were co-transfected with either the TPI1 promoter-driven reporter construct or the empty pGL3B vector, in combination with or without the c-Myc-expressing pcDNA3.1 plasmid, using polyethylenimine (PEI) as the transfection vehicle. Following a 48-h incubation period, reporter gene expression was quantified using the Dual-Luciferase Reporter Assay System (Yeasen, Cat# 11402ES60). Firefly luciferase activity, normalized to renilla luciferase, was measured to determine the relative promoter activity under different experimental conditions. All plasmids, including luciferase reporter plasmids containing either the wild-type TPI1 promoter or a mutant version with all three binding sites mutated, were provided by tsingke Biotechnology (Beijing). We performed ChIP-qPCR analysis on the J82 and UMUC-3 cell lines, adhering to the established procedure from a commercial kit (ABclonal, cat#RK20258-1). For the immunoprecipitation step, either c-Myc or non-specific IgG antibodies were utilized. After the resulting ChIP DNA was purified, we used qRT-PCR to quantify the relative abundance of targeted locations in the TPI1 gene’s promoter.

### Animal studies

All experiments involving animals received approval from the Ethics Committee of the Second Hospital of Lanzhou University (No. D2024-955). These were carried out in compliance with institutional guidelines. Four-week-old male BALB/c nude mice were sourced from the Lanzhou Veterinary Institute, a part of the Chinese Academy of Agricultural Sciences. To create subcutaneous xenograft models, 5 × 10⁶ cells were administered into the right axilla of each mouse. Monitoring of tumor growth occurred every three days through the measurement of tumor length (L) and width (W). The volume was determined using the formula (L × W²)/2. Upon the completion of each experiment (at Day 30), the mice underwent humane euthanasia via an overdose of pentobarbital injected intraperitoneally (i.p.). For evaluating the effectiveness of TPI1 knockdown, UMUC-3 cells (sh-NC or sh-TPI1) were used to inoculate the mice. The animals were then randomly allocated into four groups (n = 5 each): sh-NC + PBS, sh-TPI1 + PBS, sh-NC + GEM, and sh-TPI1 + GEM. In every experiment, treatment protocols were initiated on day 10. GEM was delivered through an intraperitoneal (i.p.) injection (at a dose of 25 mg/kg) every three days.

### Statistical analysis

Statistical analysis was conducted using GraphPad Prism software (version 8.0.0 for Windows, GraphPad Software, http://www.graphpad.com), with results presented as mean ± standard deviation (SD). Group comparisons were performed using one-way analysis of variance (ANOVA) with Tukey-Kramer post hoc testing. The Tukey-Kramer method was specifically employed to examine all possible pairwise group differences following a significant ANOVA result (p < 0.05). This approach maintains appropriate statistical power while controlling for multiple comparisons. All experiments included at least three independent replicates to ensure reproducibility.

## Results

### TPI1 modulates Gem resistance and Gem-induced apoptosis in BCa

**Fig. 1:**
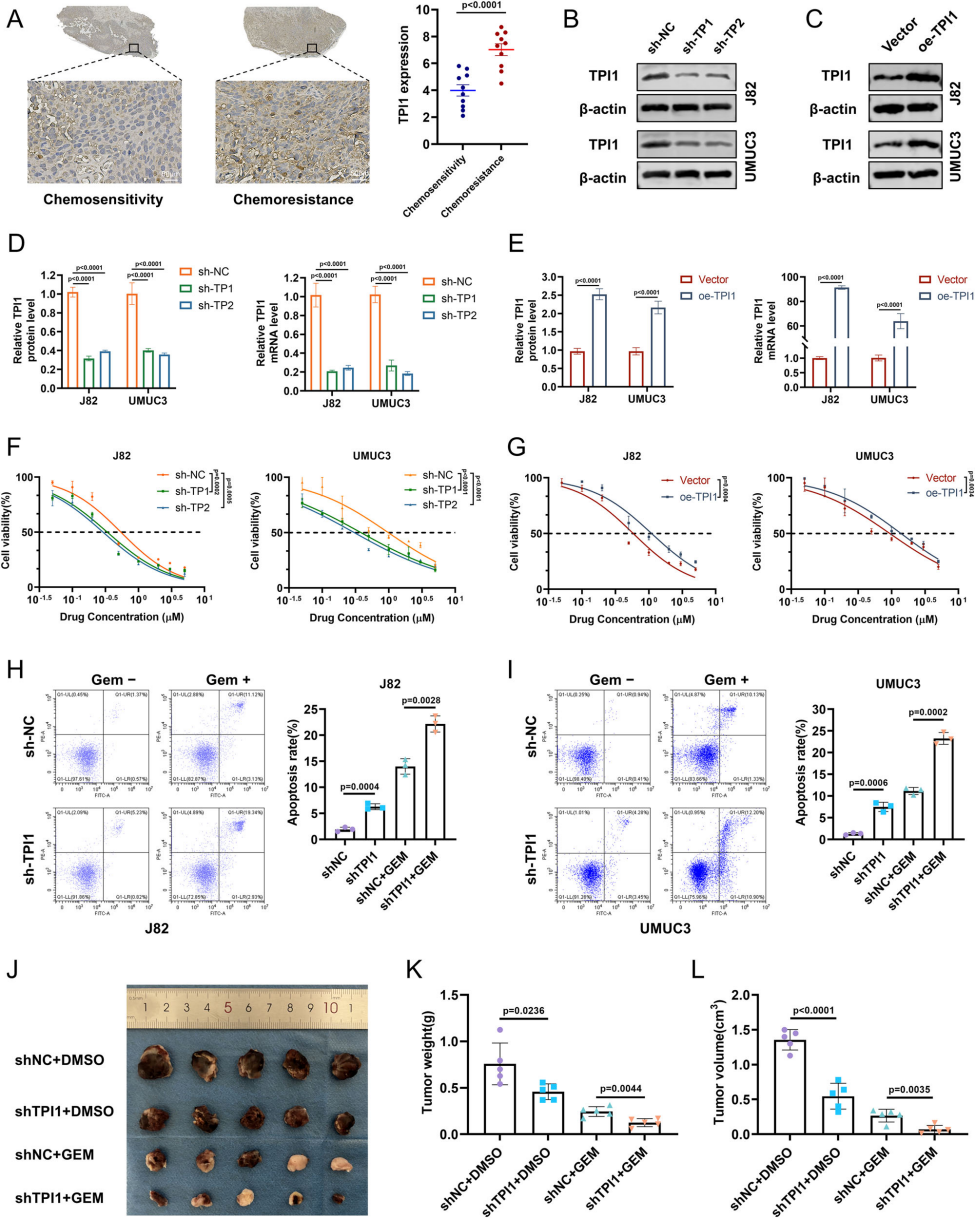
TPI1 modulates Gem resistance and Gem-induced apoptosis in BCa. **A** TPI1 expression levels in Gem-resistant and Gem-sensitive BCa patient samples. **B**, **C** Western blot analysis confirming the successful knockdown and overexpression of TPI1 in J82 and UMUC3 BCa cell lines. **D**, **E** Western blot and qPCR analysis of TPI1 knockdown and overexpression in J82 and UMUC3 cells. **F** Gem sensitivity was assessed in TPI1-knockdown J82 and UMUC3 cells treated with the indicated concentrations of Gem (0, 0.05, 0.1, 0.2, 0.5, 1, 2, 3, 5 μM) for 24 h. **G** Gem resistance was assessed in TPI1-overexpressing J82 and UMUC3 cells treated with the indicated concentrations of Gem (as in **F**) for 24 h. **H**, **I** Flow cytometry-based analysis of apoptosis in TPI1-knockdown J82 and UMUC3 cells treated with Gem (0.4 μM for J82, 0.6 μM for UMUC3) for 24 h. **J** Representative photographs of tumors from nude mice that received subcutaneous injection of BCa cells and were treated with Gem (25 mg/kg) (*n* = 5). **K** Weight of tumor-bearing bodies in each group of nude mice and analysis of their statistical differences. **L** Volume analysis of tumor-bearing bodies in nude mice of each group and statistical differences. Data are presented as mean ± SD from three independent experiments. P values in (**A**) were calculated using a two-tailed unpaired Student’s *t* test. P values in (**D**, **E**, **H**, **I**, **K**, **L**) were calculated by one-way ANOVA with Tukey’s multiple comparisons test.

TPI1 expression is significantly elevated in Gem-resistant BCa patient samples compared to Gem-sensitive counterparts (Fig. 1A). The successful knockdown and overexpression of TPI1 in BCa cell lines J82 and UMUC3 were confirmed by Western blot and qPCR experiments (Fig. 1B–E). Functional assays revealed that TPI1 knockdown significantly enhanced Gem sensitivity (Fig. 1F), whereas TPI1 overexpression promoted chemoresistance (Fig. 1G). Meanwhile, we examined the TPI1 protein levels in four distinct BCa lines: RT-4, 5637, J82, and UMUC3. The results revealed significant differences in TPI1 protein expression among the cell lines, with UMUC3 cells exhibiting the highest level and RT-4 cells the lowest (Fig. S1A and B). Subsequently, we assessed the viability of these four cell lines following treatment with Gem. Notably, the order of resistance to Gem (UMUC3 > J82 > 5637 > RT-4) was highly consistent with the protein expression levels of TPI1 (Fig. S1C). Furthermore, flow cytometry-based apoptosis assays indicated that TPI1 knockdown markedly increased Gem-induced apoptosis in both J82 and UMUC3 cells (Fig. 1H and I). To further investigate the role of TPI1 in Gem resistance in BCa in vivo, we established subcutaneous xenograft models in nude mice using control UMUC3 cells and TPI1-knockdown UMUC3 cells. The results showed that, compared to the control group, tumor volumes were significantly reduced in both the TPI1-knockdown group and the gemcitabine-treated group. More importantly, the combination of TPI1 knockdown and gemcitabine treatment led to a further reduction in tumor volume compared to gemcitabine treatment alone (Fig. 1J–L). These findings collectively underscore the pivotal role of TPI1 in regulating Gem resistance and apoptosis in BCa.

### Gem-induced autophagy is amplified under the influence of TPI1

**Fig. 2:**
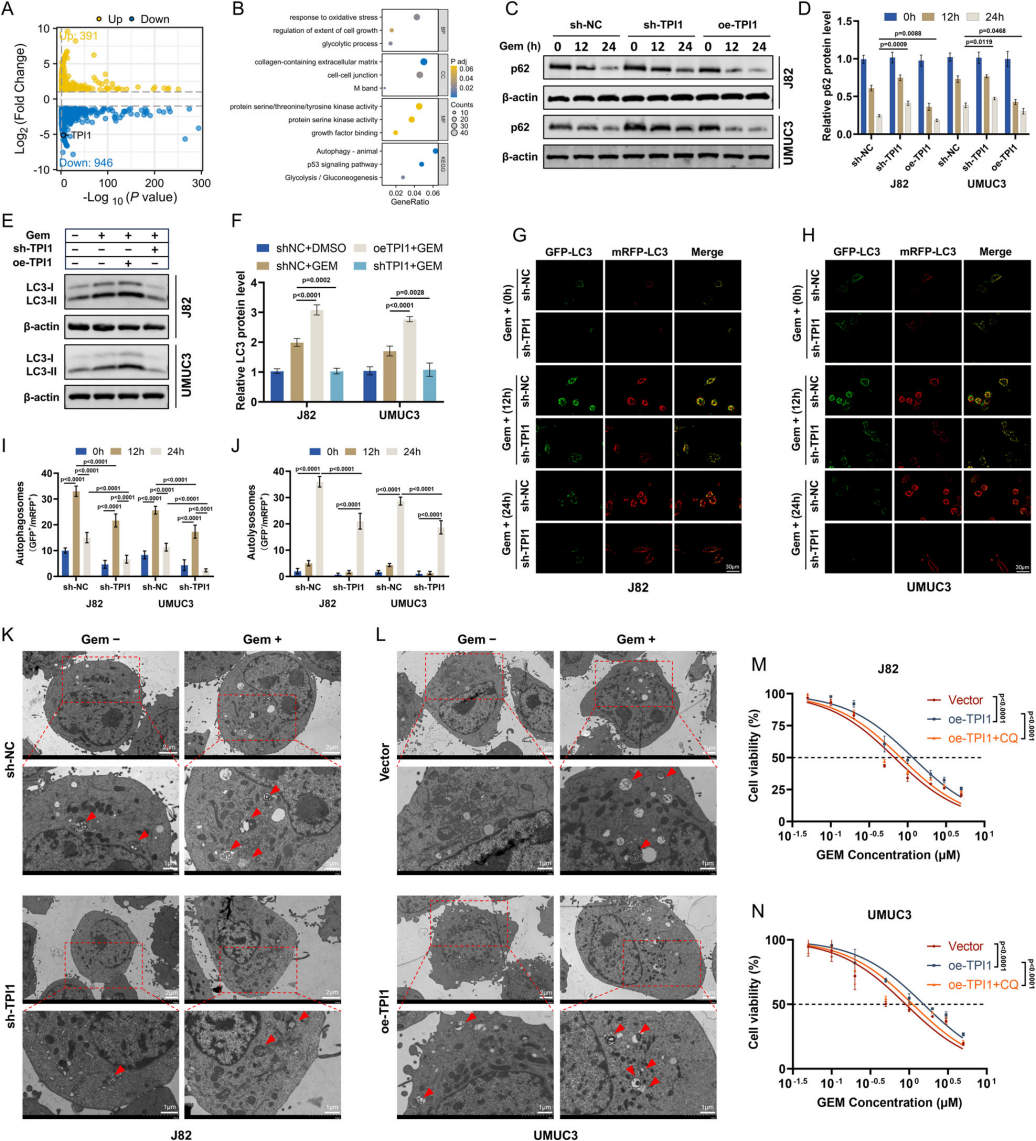
Gem-induced autophagy is amplified under the influence of TPI1. **A**, **B** Transcriptome sequencing and functional enrichment analysis of differentially expressed genes in TPI1 knockdown J82 cells treated with Gem (0.4 μM) for 24 h. **C**, **D** Western blot analysis of p62 expression in J82 and UMUC3 cells after Gem treatment (0.4 μM for J82, 0.6 μM for UMUC3). **E**, **F** Western blot analysis of LC3 expression in J82 and UMUC3 cells after Gem treatment (as in **C**) for 24 h. **G**, **H** Fluorescence microscopy images of J82 and UMUC3 cells transfected with GFP-mRFP-LC3 vector (as in **C**). Quantification of the average number of autophagosomes (**I**) and autolysosomes (**J**) per cell for the conditions described in (**G**) and (**H**). **K**, **L** Transmission electron microscopy images of J82 and UMUC3 cells following Gem treatment (as in **C**) for 24 h. Red arrows: representative images of the autophagic structures. **M**, **N** Gem Sensitivity assays of J82 and UMUC3 cells overexpressing TPI1 treated with the autophagy inhibitor chloroquine (CQ) (Gem, 0, 0.05, 0.1, 0.2, 0.5, 1, 2, 3, 5 μM; CQ, 30 μM) for 24 h. Data are presented as mean ± SD from three independent experiments. P values in (**D**, **F**, **I**, **J**) were calculated by two-way ANOVA with Tukey’s multiple comparisons test. P values in (**M**, **N**) were calculated using a two-tailed unpaired Student’s *t* test.

To investigate how TPI1 influences Gem resistance in BCa cells, we performed transcriptome sequencing on both the control group and the TPI1 knockdown group of J82 cells following Gem treatment. The analysis identified differentially expressed genes, and functional enrichment analysis revealed that TPI1 is primarily associated with autophagy, the p53 signaling pathway, and glycolysis (Fig. 2A and B). Western blot analysis was conducted to evaluate the expression of autophagy-related proteins. After Gem treatment, the expression of p62 increased in the TPI1 knockdown group compared to the control group in both J82 and UMUC3 cells. In contrast, p62 expression decreased in the TPI1 overexpression group relative to the control group (Fig. 2C and D). Furthermore, LC3-II expression was enhanced in the TPI1 overexpression group compared to the control group, while LC3-II expression was reduced in the TPI1 knockdown group (Fig. 2E and F). These findings suggest that TPI1 plays a critical role in modulating autophagy-related proteins and pathways, potentially contributing to Gem resistance in BCa cells. Further studies are warranted to elucidate the precise mechanisms underlying these observations. To monitor autophagic flux, J82 and UMUC3 cells were transduced with a dual-fluorescent GFP-mRFP-LC3 reporter construct. This system enabled the differentiation between autophagosomes (displaying yellow fluorescence due to GFP and mRFP colocalization) and autolysosomes (exhibiting red fluorescence from mRFP alone). The result revealed untreated cells (0 h) in both J82 and UMUC3 cell lines exhibited almost no LC3 fluorescent puncta. Following Gem treatment, autophagy was significantly activated in the control cells. Notably, at 12 h, an abundance of yellow puncta (autophagosomes) and red puncta (autolysosomes) were observed, indicating that autophagic flux was initiated and actively proceeding. By 24 h, the proportion of red puncta in the control cells appeared to increase compared to the 12-hour time point, suggesting that more autophagosomes had successfully fused with lysosomes, advancing to the later stages of autophagic degradation. In contrast, in the TPI1-knockdown cells, the number of yellow and red fluorescent puncta induced by Gem was markedly lower than in the control group at both 12 and 24 h (Fig. 2G and H). The quantitative analyses precisely illustrate this dynamic process (Fig. 2I and J). TEM further revealed that in J82 cells, TPI1 knockdown reduced the formation of autophagic structures induced by Gem treatment. Additionally, in UMUC3 cells, overexpression of TPI1 enhanced the autophagic structures triggered by Gem (Fig. 2K and L). Importantly, this suggests the existence of a basal level of TPI1-mediated autophagy under normal survival conditions. Although Gem treatment can enhance TPI1-activated autophagy, this is further supported by the significant increase in autophagosome numbers in high-TPI1-expressing cells following Gem treatment. Meanwhile, in both J82 and UMUC3 cells overexpressing TPI1, treatment with the autophagy inhibitor chloroquine (CQ) enhanced the sensitivity to Gem (Fig. 2M and N).

### TPI1 regulates Gem sensitivity by affecting autophagy in drug-resistant strains

**Fig. 3:**
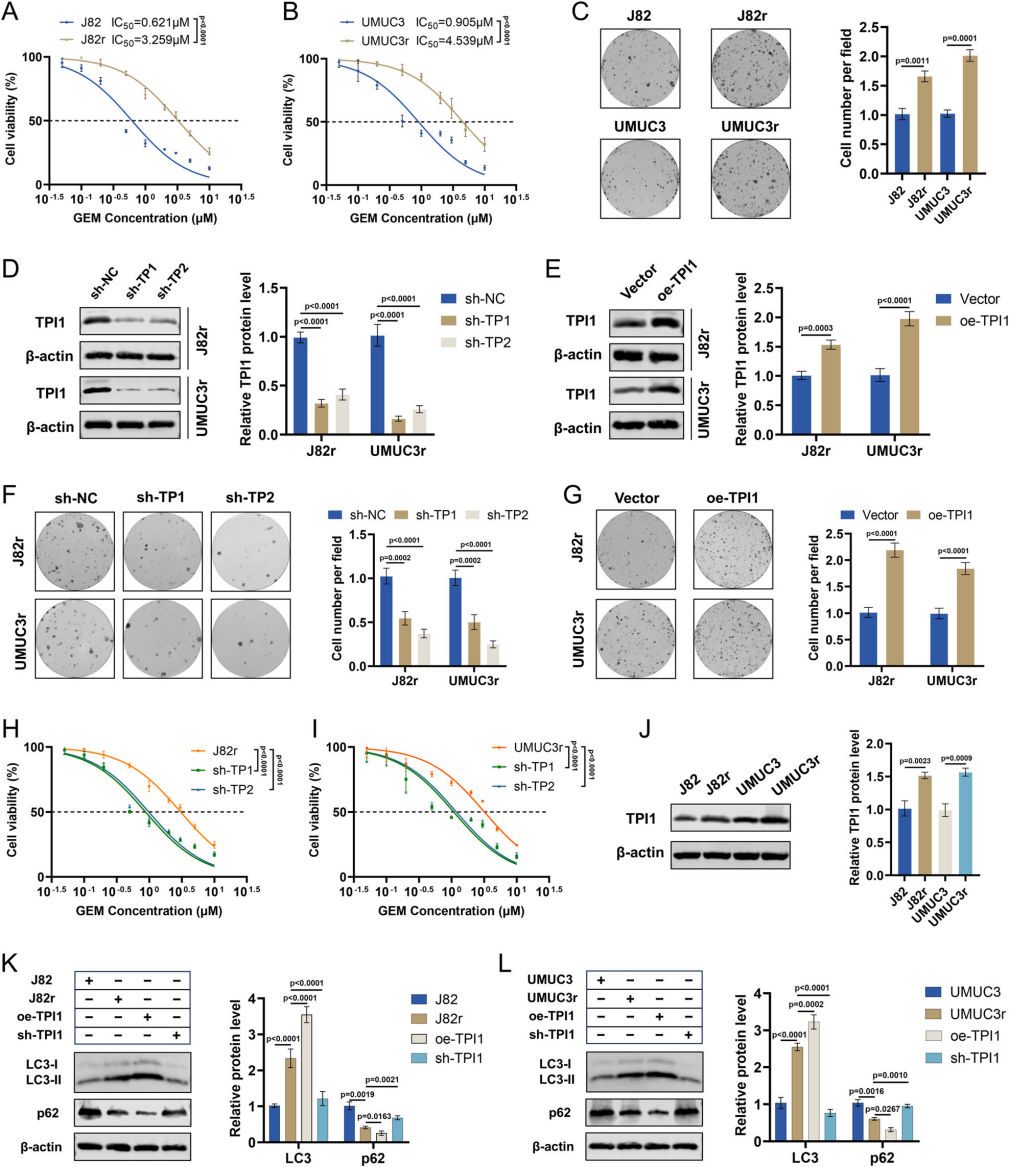
TPI1 regulates Gem sensitivity by affecting autophagy in drug-resistant strains. **A**, **B** Establishment of Gem-resistant strains J82r and UMUC3r derived from J82 and UMUC3 cells, respectively (Gem, 0, 0.05, 0.1, 0.2, 0.5, 1, 2, 3, 5, 10 μM). **C** Colony formation assays assessing the proliferative capacity of J82r and UMUC3r cells compared to their parental cells after treatment with Gem (1.2 μM for J82r, 2 μM for UMUC3r) for 24 h (left) and quantification (right). **D**, **E** Construction of stable TPI1-knockdown and TPI1-overexpressing cell lines using J82r and UMUC3r (left) and quantification (right). **F**, **G** Colony formation assays assessing the proliferative ability of J82r and UMUC3r cells with TPI1 knockdown or overexpression under Gem treatment (as in **C**) (left) and quantification (right). **H**, **I** CCK-8 assays showing that TPI1 knockdown in J82r and UMUC3r significantly increased sensitivity to Gem. **J** Western blot analysis of TPI1 protein levels in parental (J82, UMUC3) and their corresponding resistant (J82r, UMUC3r) cell lines (left) and quantification (right). **K**, **L** Western blot analysis of LC3-II and p62 expression in J82r and UMUC3r cells (left) and quantification (right). Data are presented as mean ± SD from three independent experiments. P values in (**A**, **B**, **C**, **E**, **G**, **J**) were calculated using a two-tailed unpaired Student’s *t* test. P values in (**D**, **F**, **H**, **I**, **K**, **L**) were calculated by one-way ANOVA with Tukey’s multiple comparisons test.

To further validate the impact of TPI1 on Gem sensitivity in BCa cells, we established Gem-resistant strains, J82r and UMUC3r, derived from J82 and UMUC3 cells, respectively (Fig. 3A and B). Colony formation assays demonstrated that after 24 h of Gem treatment, J82r and UMUC3r exhibited stronger proliferative capacity compared to J82 and UMUC3 cells (Fig. 3C). Subsequently, we constructed stable TPI1 knockdown and TPI1-overexpressing cell lines using J82r and UMUC3r (Fig. 3D and E). The colony formation assay showed that after Gem treatment, the proliferation ability of J82r and UMUC3r cells with TPI1 knockdown was significantly weaker than that of the control group, while overexpression of TPI1 markedly enhanced their proliferation ability (Fig. 3F and G). CCK-8 assays revealed that the knockdown of TPI1 in J82r and UMUC3r significantly increased sensitivity to Gem, further confirming the role of TPI1 in modulating Gem sensitivity in BCa cell lines (Fig. 3H and I). To investigate the expression of TPI1 in drug-resistant BCa cells, we examined its protein levels in the parental cell lines (J82, UMUC3) and their corresponding resistant counterparts (J82r, UMUC3r). The results clearly showed that TPI1 protein expression was markedly upregulated in the resistant J82r and UMUC3r cell lines compared to the parental cells (Fig. 3J). Western blot analysis showed that compared to J82 and UMUC3 cells, the expression of LC3-II was elevated, while the expression of p62 was reduced in J82r and UMUC3r cells, indicating that autophagy is involved in enhancing Gem resistance in BCa cells (Fig. 3K and L). In J82r and UMUC3r cells, overexpression of TPI1 led to increased LC3-II expression and decreased p62 expression, whereas knockdown of TPI1 resulted in reduced LC3-II expression and increased p62 expression. These findings suggest that TPI1 contributes to Gem resistance in BCa cells by regulating autophagy.

### TPI1 modulates autophagy through its interaction with Beclin-1

**Fig. 4:**
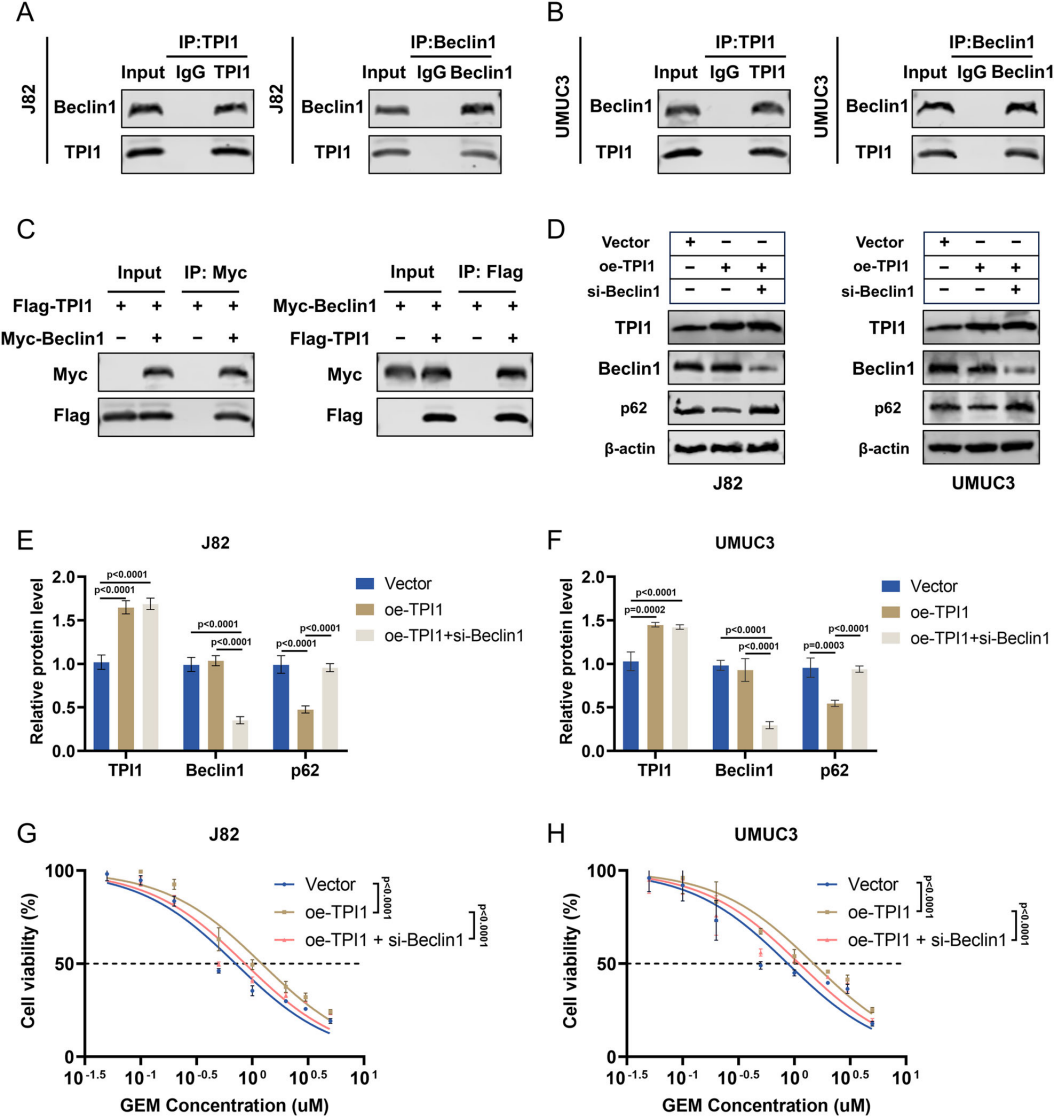
TPI1 modulates autophagy through its interaction with Beclin-1 in BCa cells. **A**, **B** Endogenous Co-IP of TPI1 and Beclin-1 in J82 and UMUC3 cells. **C** Exogenous Co-IP of TPI1 and Beclin-1 in cells transfected with TPI1 and Beclin-1 expression plasmids. **D**–**F** Western blot analysis of Beclin-1 and p62 expression in BCa cells overexpressing TPI1 with or without Beclin-1 knockdown and quantification (Gem, 0.4 μM for J82, 0.6 μM for UMUC3). **G**, **H** CCK-8 assays show that knockdown of Beclin-1 attenuates the enhanced Gem resistance induced by TPI1 overexpression (Gem, 0, 0.05, 0.1, 0.2, 0.5, 1, 2, 3, 5 μM). Data are presented as mean ± SD from three independent experiments. P values in (**E**, **F**) were calculated by two-way ANOVA with Tukey’s multiple comparisons test. P values in (**G**, **H**) were calculated using a two-tailed unpaired Student’s *t* test.

These findings suggest that TPI1 enhances Gem resistance in BCa cells by promoting autophagy. To explore how TPI1 regulates autophagy in BCa cells, we performed immunoprecipitation followed by mass spectrometry (IP-MS) analysis. This analysis identified 234 potential TPI1-binding proteins (Table S5). Our IP-MS analysis identified both Beclin-1 and VPS34 as potential TPI1-interacting proteins. We prioritized Beclin-1 for further study as it ranked as a higher-confidence candidate in our proteomic data. Functionally, Beclin-1’s role as the central regulatory scaffold for autophagy initiation makes it a more logical target for upstream regulation than the catalytic subunit VPS34. Subsequent endogenous and exogenous co-immunoprecipitation experiments confirmed that TPI1 and Beclin-1 could be co-precipitated with magnetic beads (Fig. 4A–C). To validate whether TPI1 influences autophagy through its interaction with Beclin-1, we knocked down Beclin-1 in BCa cells overexpressing TPI1. Western blot results demonstrated that overexpression of TPI1 did not significantly alter Beclin-1 expression levels but reduced p62 expression (Fig. 4D–F). However, knockdown of Beclin-1 reversed the decrease in p62 expression, indicating that TPI1 modulates autophagy in BCa cells through its interaction with Beclin-1. Further CCK-8 assays also confirmed that knockdown of Beclin-1 attenuated the enhanced Gem resistance induced by TPI1 overexpression (Fig. 4G and H).

### TPI1 enhances Gem-induced autophagy by competing with Bcl-2 for Beclin-1 binding

**Fig. 5:**
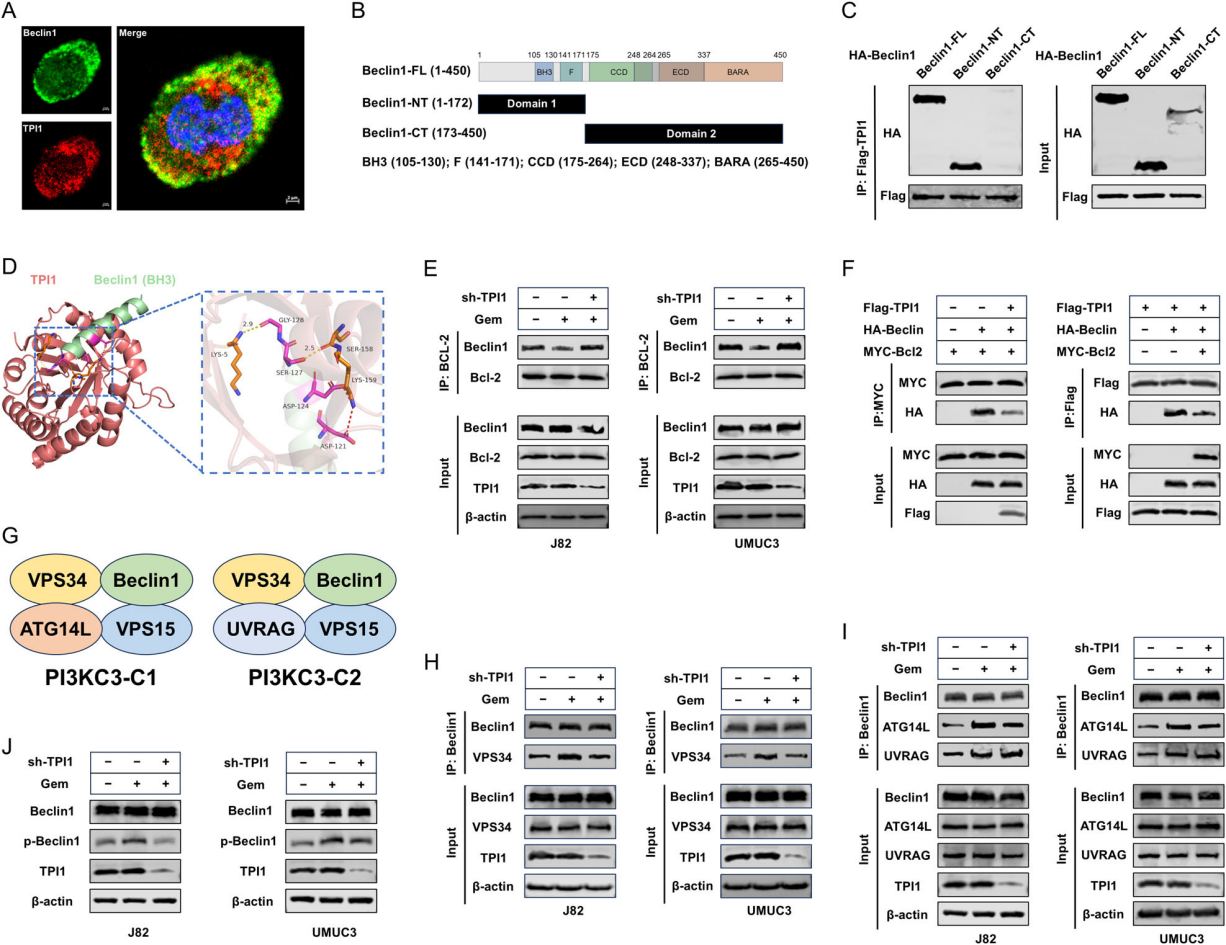
TPI1 enhances Gem-induced autophagy by competing with Bcl-2 for Beclin-1 binding. **A** Immunofluorescence co-localization analysis shows TPI1 and Beclin-1 co-localized in the cytoplasm of J82 cells. **B** Schematic representation of the truncated forms of Beclin-1: Beclin-1-NT (1-172) and Beclin-1-CT (173-450). **C** Co-IP experiments identify the interaction site between TPI1 and Beclin-1 within the Beclin-1-NT region. **D** Molecular docking models reveal multiple binding sites between TPI1 and the BH3 domain of Beclin-1. **E** Co-IP experiments in BCa cells show that Gem treatment (0.4 μM for J82, 0.6 μM for UMUC3) weakens the interaction between Beclin-1 and Bcl-2, which is reversed by TPI1 knockdown. **F** Exogenous Co-IP in 293 T cells demonstrates that TPI1 overexpression reduces the interaction between Beclin-1 and Bcl-2. **G** Schematic diagram of PIK3C3-C1 (class III PIK3 complex I) and PIK3C3-C2 (class III PIK3 complex II) composition. **H** Co-IP assays in BCa cells demonstrated that Gem treatment (as in **E**) enhanced the interaction between Beclin-1 and VPS34, while TPI1 knockdown attenuated this effect. **I** Co-IP assays in BCa cells demonstrated that Gem treatment (as in **E**) enhanced interactions between Beclin-1 and both ATG14L and UVRAG, while TPI1 knockdown specifically attenuated the Beclin-1/ATG14L interaction. **J** In BCa cells, Gem treatment (as in **E**) enhanced Beclin-1 phosphorylation, while TPI1 knockdown attenuated this phosphorylation event.

To investigate how the interaction between TPI1 and Beclin-1 promotes Gem-induced autophagy in BCa cells, we first performed immunofluorescence co-localization analysis and found that TPI1 and Beclin-1 co-localized in the cytoplasm of J82 cells (Pearson_Rr = 0.73) (Figs. 5A and S2A). To identify the interaction domains between TPI1 and Beclin-1, we generated two truncated forms of Beclin-1 based on previous studies: Beclin-1-NT (1-172) and Beclin-1-CT (173-450) (Fig. 5B). The results indicated that the interaction site with TPI1 lies within the structural threshold of the Beclin-1-NT region (Fig. 5C). Molecular docking models of TPI1 and Beclin-1 revealed multiple binding sites between TPI1 and the BH3 domain of Beclin-1 (Fig. 5D). The BH3 domain is a critical region of the Beclin-1 protein and serves as the binding site for Bcl-2. Under normal conditions, Bcl-2 binds to the BH3 domain of Beclin-1, thereby inhibiting Beclin-1-induced autophagy. However, under stress conditions, this interaction dissociates, allowing Beclin-1 to promote autophagy. In BCa cells, we observed through co-immunoprecipitation experiments that the binding between Beclin-1 and Bcl-2 was weakened under Gem treatment. However, when TPI1 was knocked down, this weakening was not significantly observed (Figs. 5E and S2B and C). Further exogenous immunoprecipitation results in 293 T cells showed that, compared to the untransfected Flag-TPI1 group, the HA-Beclin-1 band was significantly reduced in the Flag-TPI1-transfected group, indicating a weakened interaction between MYC-Bcl2 and HA-Beclin-1. Similarly, compared to the untransfected MYC-Bcl2 plasmid group, the HA-Beclin-1 band was significantly reduced, suggesting a weakened interaction between Flag-TPI1 and HA-Beclin-1 (Figs. 5F and S2D and E). In mammals, Beclin-1 serves as a core component of two distinct class III phosphatidylinositol 3-kinase (PIK3C3) complexes: PIK3C3-C1 (PIK3C3 complex I, containing Beclin-1, VPS34, VPS15, and ATG14L) and PIK3C3-C2 (PIK3C3 complex II, comprising Beclin-1, VPS34, VPS15, and UVRAG) (Fig. 5G). These complexes play essential roles in mediating the formation and maturation of autophagosomes and endocytic vesicles, making Beclin-1 an indispensable factor for autophagy initiation [19–23]. To determine whether TPI1 promotes the formation of Beclin-1-associated PIK3C3 complexes, we performed co-immunoprecipitation assays and found that TPI1 knockdown attenuated the Gem-induced increase in Beclin-1/VPS34 binding (Figs. 5H and S2F and G). To further identify which PIK3C3 complex is regulated by TPI1, we observed that TPI1 knockdown significantly reduced the interaction between Beclin-1 and ATG14L (Figs. 5I and S2H and I), indicating that TPI1 primarily facilitates the assembly of PIK3C3-C1, thereby promoting autophagy. The ULK1 complex phosphorylates Beclin-1 at Ser15 in its N-terminal region, which subsequently activates PIK3C3-C1 to promote autophagy induction. We found that TPI1 knockdown significantly reduced phosphorylation of Beclin-1 at Ser15, further confirming that TPI1 primarily regulates the formation of PIK3C3-C1 (Figs. 5J and S2J and K).

### TPI1 enhances Gem-induced mitophagy in BCa cells

**Fig. 6:**
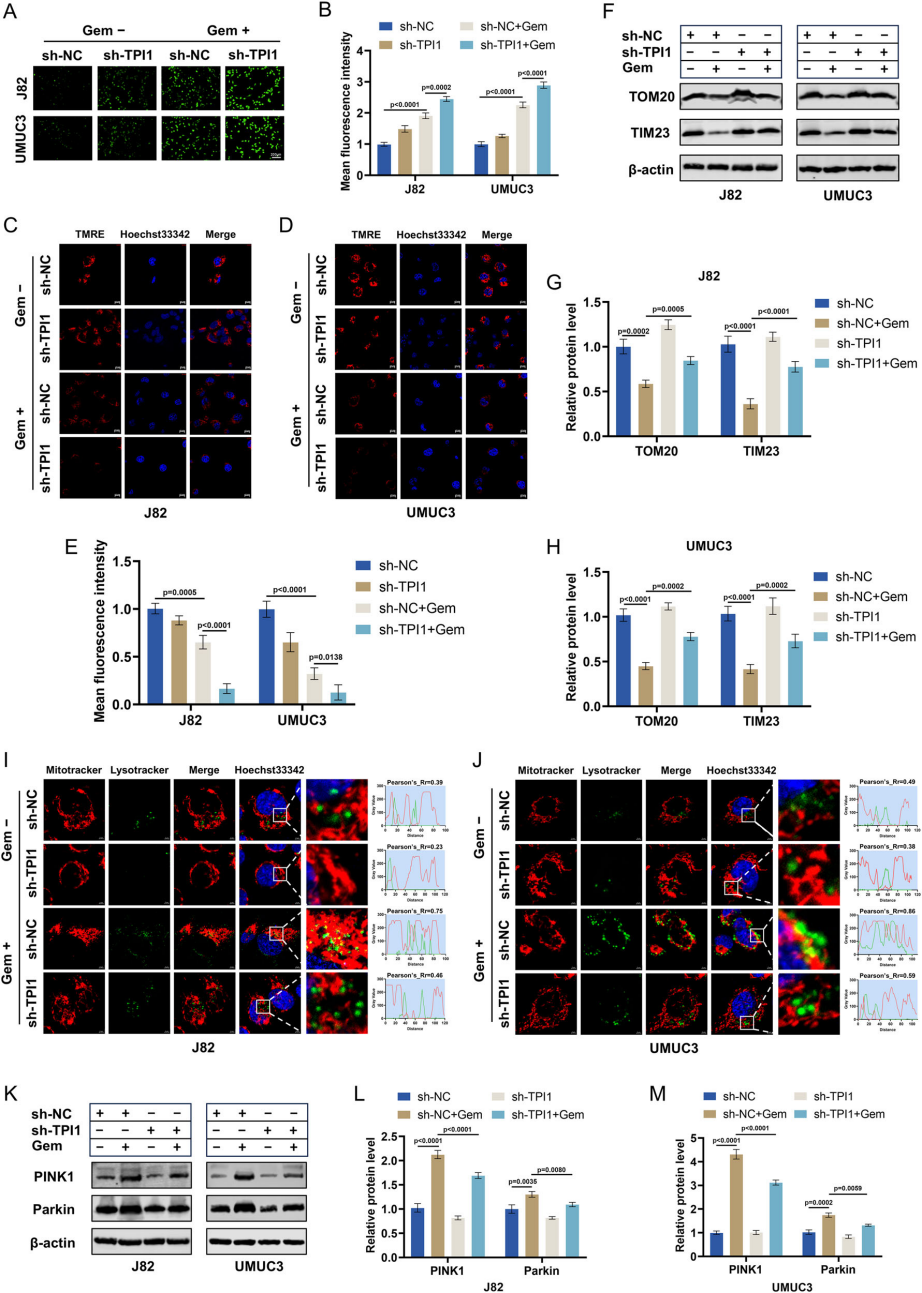
TPI1 enhances Gem-induced mitophagy in BCa cells. **A**, **B** DCFH-DA staining shows intracellular ROS levels in J82 and UMUC3 cells with or without TPI1 under Gem treatment (0.4 μM for J82, 0.6 μM for UMUC3) and quantification. **C**–**E** TMRE staining assesses mitochondrial membrane potential in J82 and UMUC3 cells with or without TPI1 under Gem treatment (as in **A**) and quantification. **F**–**H** Western blot analysis of mitochondrial-specific proteins TOM20 and TIM23 in BCa cells with or without TPI1 under Gem treatment (as in **A**) and quantification. **I**, **J** Immunofluorescence analysis of mitochondrial and lysosomal co-localization in J82 and UMUC3 cells with or without TPI1 under Gem treatment (as in **A**). **K**–**M** Western blot analysis of PINK1 and Parkin protein levels in control and TPI1-knockdown J82 and UMUC3 cells, treated with or without Gem (as in **A**) and quantification. Data are presented as mean ± SD from three independent experiments. P values in (**B**, **E**, **G**, **H**, **L**, **M**) were calculated by one-way ANOVA with Tukey’s multiple comparisons test.

Chemotherapeutic agents such as Gem exert their effects on tumor cells by damaging mitochondria, leading to excessive ROS production, which further exacerbates DNA damage and induces apoptosis. Autophagy can clear damaged mitochondria, maintain intracellular ROS redox homeostasis, and suppress DNA damage. Therefore, we aimed to investigate whether TPI1-mediated autophagy can eliminate damaged mitochondria and maintain intracellular ROS homeostasis under Gem treatment. We used DCFH-DA staining to determine ROS levels in J82 and UMUC3 cells with or without TPI1. We found that under Gem treatment, silencing TPI1 led to increased intracellular ROS levels (Fig. 6A and B). Mitophagy can clear damaged mitochondria with low membrane potential induced by chemotherapeutic agents, thereby maintaining mitochondrial quality control and normal mitochondrial function. We used TMRE staining to assess mitochondrial health in J82 and UMUC3 cells with or without TPI1. We observed that, compared to the control, silencing TPI1 under Gem treatment resulted in an increase in damaged mitochondria with low membrane potential (Fig. 6C–E). Further experiments confirmed through Western blot that silencing TPI1 inhibited the reduction in the expression of mitochondrial-specific proteins TOM20 and TIM23 induced by Gem in BCa cells (Fig. 6F–H). This finding further supports that silencing TPI1 in J82 and UMUC3 cells suppresses the clearance of Gem-induced mitochondrial damage. Additionally, TPI1 knockdown in J82 and UMUC3 cells reduced the co-localization of mitochondria and lysosomes induced by Gem, indicating that TPI1 knockdown inhibits Gem-induced mitophagy in BCa cells (Fig. 6I and J). To investigate the underlying mechanism of TPI1-mediated mitophagy, we measured PINK1 and Parkin protein levels in TPI1-knockdown and control J82 and UMUC3 cells, both before and after Gem treatment. In control cells, Gem treatment led to a significant upregulation of PINK1 and Parkin, indicating the induction of PINK1/Parkin-dependent mitophagy. Notably, this Gem-induced upregulation was substantially attenuated in TPI1-knockdown cells compared to controls (Fig. 6K–M). These results suggest that TPI1 depletion hinders the activation of the PINK1/Parkin pathway in response to Gem, indicating that TPI1 may regulate chemosensitivity in BCa cells through its role in mitophagy.

### TPI1 enhances Gem-induced mitophagy in BCa tissues

**Fig. 7:**
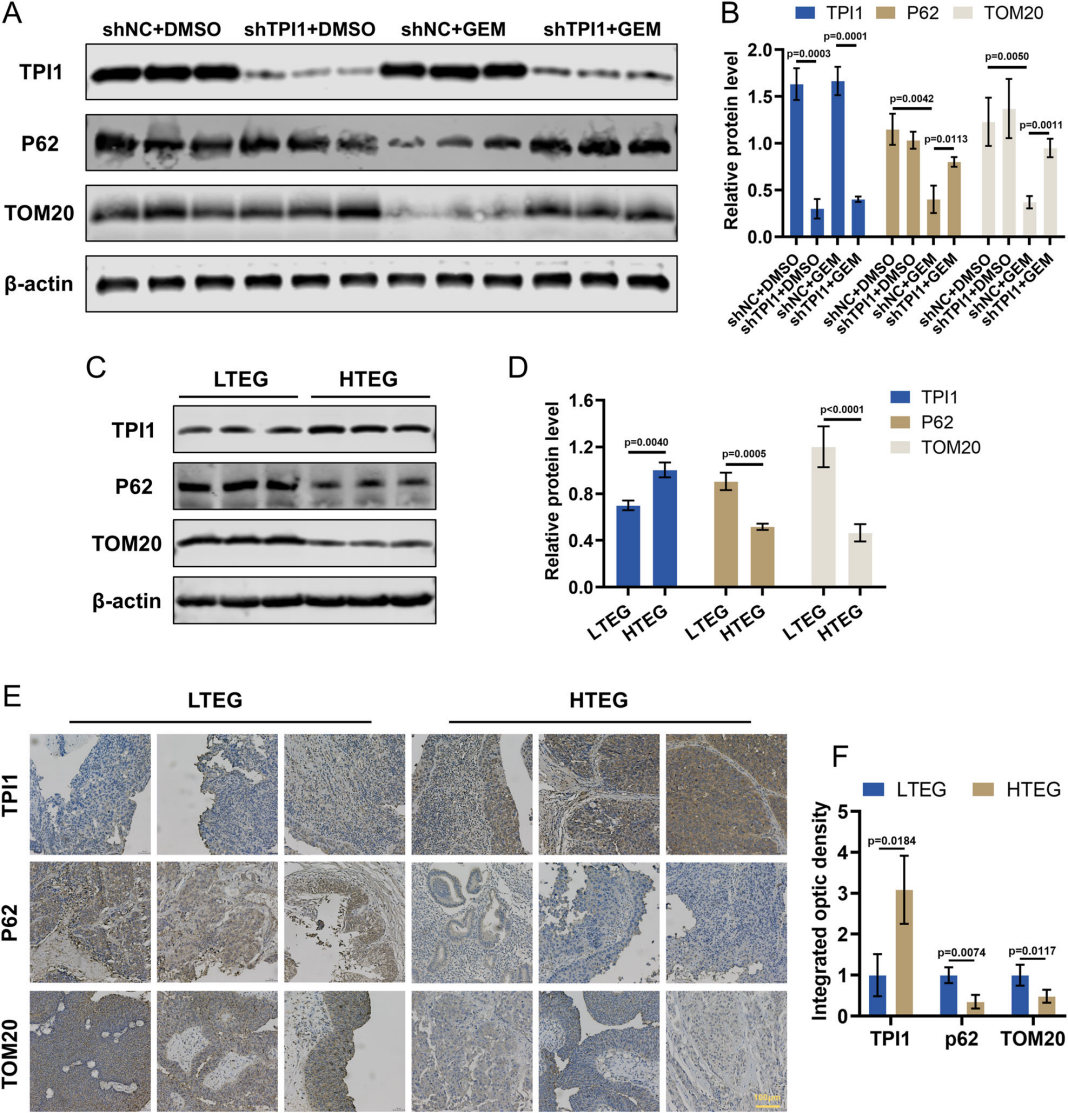
TPI1 enhances Gem-induced mitophagy in BCa tissues. **A**, **B** Western blot analysis of p62 and TOM20 expression in subcutaneous tumor tissues from different groups of nude mice and quantification. **C**, **D** Western blot analysis of p62 and TOM20 expression in chemotherapy-resistant human BCa tissues grouped into high TPI1 expression (HTEG) and low TPI1 expression (LTEG) and quantification. **E**, **F** Immunohistochemical staining of p62 and TOM20 in HTEG and LTEG and quantification. Data are presented as mean ± SD from three independent experiments. P values in (**B**) were calculated by one-way ANOVA with Tukey’s multiple comparisons test. P values in (**D**, **F**) were calculated using a two-tailed unpaired Student’s *t* test.

The above findings confirm that TPI1 influences Gem-induced mitophagy in BCa cells. In subcutaneous xenograft tissues of nude mice, Western blot analysis revealed that compared with the control group, the gemcitabine-treated group exhibited significant downregulation of p62 and TOM20 expression. However, the combination of TPI1 knockdown with gemcitabine treatment attenuated this downregulation of p62 and TOM20 (Fig. 7A and B). To further explore the relationship between TPI1 and mitophagy in human BCa tissues, we collected samples from chemotherapy-resistant patients and divided them into high TPI1 expression groups (HTEG) and low TPI1 expression groups (LTEG) based on TPI1 expression levels. Western blot analysis revealed that the expression of p62 and TOM20 was significantly lower in the HTEG compared to the LTEG (Fig. 7C and D). Further immunohistochemical results also demonstrated that the expression of p62 and TOM20 was significantly reduced in the HTEG compared to the LTEG (Fig. 7E and F). These findings indicate that TPI1-mediated enhancement of mitophagy is positively correlated with Gem chemotherapy resistance in human BCa patients.

### c-Myc transcriptionally activates TPI1 to promote Gem resistance in BCa

**Fig. 8:**
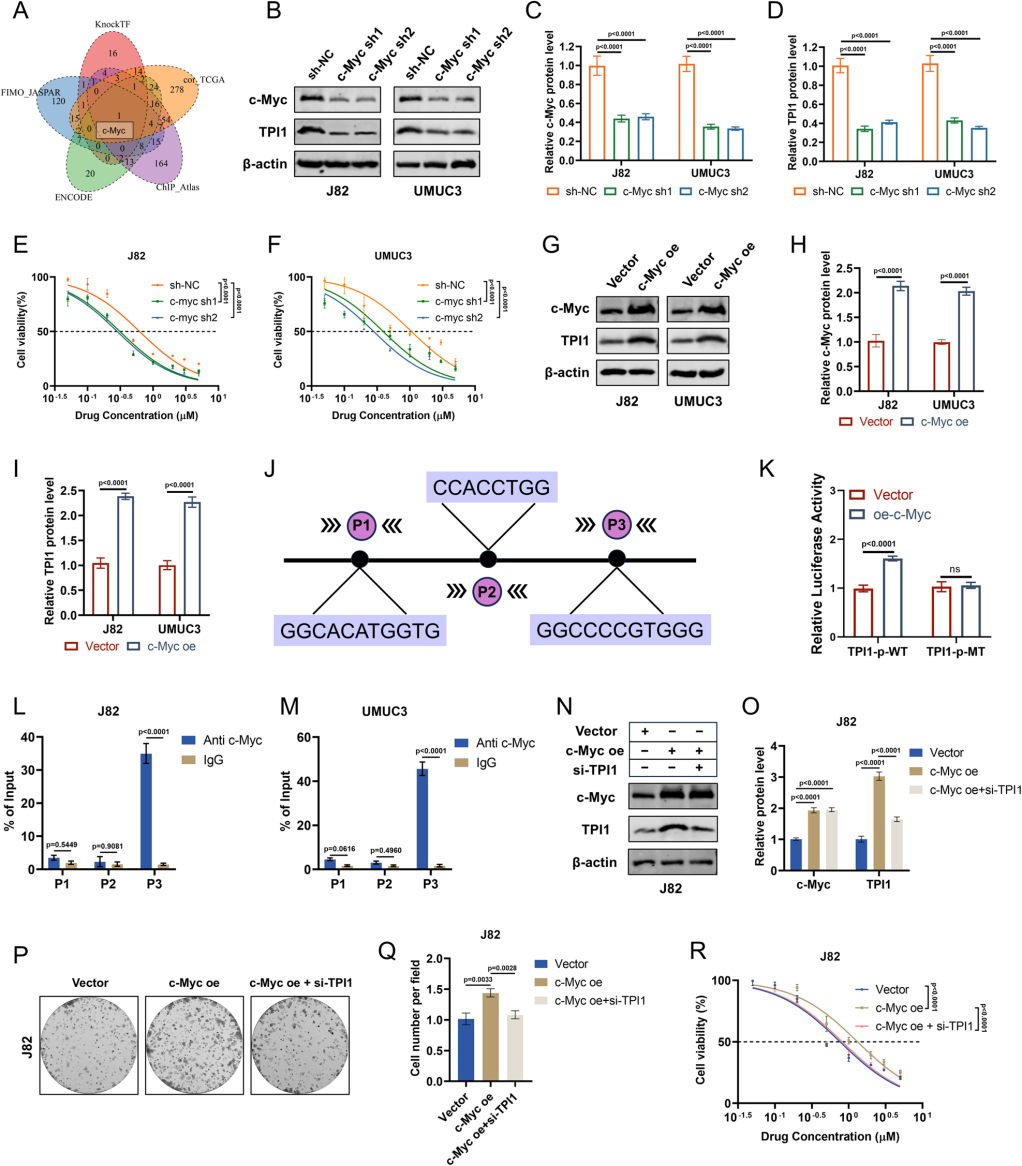
c-Myc transcriptionally activates TPI1 to promote Gem resistance in BCa. **A** Identification of c-Myc as a potential transcription factor regulating TPI1 using four TF prediction network tools and correlation analysis in the TCGA-BLCA dataset. **B**, **C** c-Myc knockdown models in J82 and UMUC3 cells. Knockdown efficiency was confirmed by Western blot. **D** Western blot analysis of TPI1 protein expression in c-Myc knockdown cells. **E**, **F** Gem sensitivity assays in c-Myc knockdown J82 and UMUC3 cells. **G**, **H** c-Myc overexpression models in J82 and UMUC3 cells. Overexpression efficiency was confirmed by Western blot. **I** Western blot analysis of TPI1 protein expression in c-Myc-overexpressing cells. **J** Prediction of three potential c-Myc binding sites in the TPI1 promoter using the JASPAR database. **K** Dual-luciferase reporter assay analyzing the regulation of TPI1 promoter activity by c-Myc using reporter plasmids containing either the wild-type TPI1 promoter (TPI1-p-WT) or its binding site-mutated version (TPI1-p-MT). Chromatin immunoprecipitation-quantitative PCR (ChIP-qPCR) analysis assessing the binding of endogenous c-Myc protein to the TPI1 promoter region in J82 (**L**) and UMUC3 (**M**) cells. **N**, **O** TPI1 knockdown in c-Myc-overexpressing J82 cells. Knockdown efficiency was confirmed by Western blot and quantification. **P**, **Q** Colony formation assays show that TPI1 knockdown rescues the c-Myc overexpression-induced promotion of cell proliferation and quantification. **R** TPI1 knockdown reverses the Gem resistance caused by c-Myc overexpression. Data are presented as mean ± SD from three independent experiments. P values in (**C**, **D**, **O**, **Q**) were calculated by one-way ANOVA with Tukey’s multiple comparisons test. P values in (**E**, **F**, **H**, **I**, **K**, **L**, **M**, **R**) were calculated using a two-tailed unpaired Student’s *t* test.

To explore the upstream regulatory mechanisms of TPI1 more comprehensively, we further analyzed potential transcription factors (TFs) that might regulate TPI1. Based on the results from four TF prediction network tools and correlation analysis of TPI1 in the TCGA-BLCA dataset, a cross-referenced gene, c-Myc, was identified (Fig. 8A). In this study, we constructed c-Myc knockdown models in J82 and UMUC3 cells (Fig. 8B and C). Compared to the control group, the expression of TPI1 protein was significantly reduced in c-Myc knockdown cells (Fig. 8D). Additionally, c-Myc knockdown in BCa cells significantly enhanced Gem sensitivity (Fig. 8E and F). Furthermore, c-Myc overexpression models were established in J82 and UMUC3 cells (Fig. 8G and H). Compared to the control group, TPI1 protein expression was significantly increased in c-Myc-overexpressing cells (Fig. 8I). To determine whether c-Myc binds to the binding sites in the TPI1 promoter, three potential binding sites were predicted using the JASPAR database (Fig. 8J). To functionally validate the transcriptional regulation of the TPI1 promoter by c-Myc, we constructed luciferase reporter plasmids containing either the wild-type TPI1 promoter (TPI1-p-WT) or a mutant version with all three binding sites mutated (TPI1-p-MT). In the presence of TPI1-p-WT, overexpression of c-Myc resulted in a highly significant increase in luciferase activity compared to the empty vector control group, indicating that c-Myc potently activates the TPI1 promoter. However, when these critical sites in the TPI1 promoter were mutated (TPI1-p-MT), this transcriptional activation by c-Myc was completely abolished (Fig. 8K). To validate the direct interaction between the c-Myc protein and the TPI1 promoter in vivo, we performed ChIP-qPCR assays in J82 (Fig. 8L) and UMUC3 (Fig. 8M) cells. The results clearly demonstrated that the c-Myc protein specifically binds to the P3 site on the TPI1 promoter. To further validate the regulatory relationship, we knockdown TPI1 in c-Myc-overexpressing BCa cells (Fig. 8N and O). Colony formation assays revealed that TPI1 knockdown rescued the c-Myc overexpression-induced promotion of cell proliferation (Fig. 8P and Q). At the same time, TPI1 knockdown reversed the Gem resistance caused by c-Myc overexpression (Fig. 8R). Furthermore, we found that compared to chemosensitive tumor tissues, chemoresistant tissues exhibited significantly higher staining intensity and a greater proportion of c-Myc positive cells (Fig. S3A). Consistent with this, c-Myc protein levels were also significantly upregulated in the two resistant cell lines, J82r and UMUC3r, compared to their parental counterparts, J82 and UMUC3 (Fig. S3B). These results demonstrate that c-Myc transcriptionally activates TPI1, which in turn promotes Gem resistance in BCa.

## Discussion

This study comprehensively elucidated the role of TPI1 in Gem resistance in BCa cells. Clinical sample analysis and cellular experiments revealed that high TPI1 levels are associated with Gem resistance, primarily through the promotion of autophagy. As a key enzyme in glycolysis, TPI1 competitively binds to the BH3 domain of Beclin-1, thereby inhibiting the negative regulatory effect of Bcl-2 on Beclin-1. This interaction enhances autophagy in BCa cells, ultimately contributing to increased Gem resistance. Furthermore, these effects of TPI1 are positively transcriptionally regulated by c-Myc. These findings highlight the role of TPI1 in Gem resistance in BCa and suggest that it may serve as a potential target for enhancing chemotherapy efficacy in BCa.

TPI1 is a protein-coding gene located on the short arm of chromosome 12 (12p13.31). It is also known as methylglyoxal synthase, TPI, TIM, or TPID. This gene plays a crucial role in glycolysis and gluconeogenesis. It facilitates the conversion of dihydroxyacetone phosphate to glyceraldehyde 3-phosphate, ensuring smooth energy production through glycolysis or oxidative phosphorylation [24–27]. Studies have shown that TPI1 is associated with various cancers. For example, in esophageal cancer, high levels of TPI1 autoantibodies in the blood can aid in early disease detection [28]. Similar findings have been observed in lung cancer. Research has demonstrated that serum autoantibody levels are significantly higher in patients with lung adenocarcinoma and squamous cell carcinoma compared to healthy individuals. These findings help distinguish lung cancer from non-lung cancers [29, 30]. In liver cancer, proteomic analysis has revealed that TPI1 overexpression is associated with recurrence and poor outcomes in intrahepatic cholangiocarcinoma [31]. Additionally, studies have linked TPI1 to chemotherapy resistance. Notably, significant nuclear accumulation of TPI1 has been observed in lung cancer tissues, and TPI1 nuclear translocation is induced by extracellular stress, such as chemotherapy drugs, which enhances chemoresistance in lung cancer cells [32]. Our study found that TPI1 expression is elevated in chemotherapy-resistant BCa tissues. By overexpressing or knocking down TPI1 in BCa cells, we discovered that TPI1 can influence the sensitivity of these cells Gem. Furthermore, flow cytometry experiments revealed that TPI1 knockdown attenuates Gem-induced apoptosis in BCa cells.

The autophagy mechanism is regulated by autophagy-related (ATG) genes and proteins. In 1999, research by Levine’s team demonstrated that Beclin-1 is the mammalian ortholog of yeast Atg6 and is a critical component for autophagy initiation [33]. In mammalian systems, Beclin-1 serves as a crucial component in two distinct PIK3C3 complexes: complex I (PIK3C3-C1) and complex II (PIK3C3-C2). These complexes, composed of Beclin-1, VPS34, VPS15, and either ATG14 or UVRAG, respectively, play pivotal roles in both autophagosome biogenesis/maturation and endocytic processes [19–23]. The functional regulation of Beclin-1 is mediated through multiple protein interactions. Specifically, its CCD domain acts as a structural scaffold for PIK3C3 complex assembly. The competitive binding of ATG14L and UVRAG to the CCD domain differentially activates VPS34 kinase activity, leading to the formation of either PIK3C3-C1 or PIK3C3-C2 [34, 35]. Furthermore, Bcl-2 family proteins (including Bcl-2, Bcl-B, Bcl-XL, and Mcl-1) interact with the BH3 domain of Beclin-1, thereby preventing its association with ATG14L/UVRAG and subsequently inhibiting autophagy initiation [36–38]. Additionally, the Rubicon protein exerts negative regulatory effects on autophagy through BARA domain-mediated interaction with Beclin-1, resulting in decreased VPS34 activity [39]. In this study, transcriptome sequencing and cellular experiments revealed that TPI1 primarily influences Gem resistance by promoting autophagy. Further experiments, including mass spectrometry and co-immunoprecipitation, demonstrated that TPI1 competitively binds to the BH3 domain of Beclin-1, rescuing the inhibitory effect of Bcl-2 on Beclin-1. This interaction enhances the formation of the PIK3C3-C1 complex, promoting the initiation of autophagy and consequently leading to increased gemcitabine resistance in BCa cells.

The mitochondrial electron transport chain (ETC) generates ROS as a byproduct of its normal function [40]. Stimuli such as chemotherapy drugs can cause mitochondrial damage, leading to dysfunction of the ETC, abnormal mitochondrial membrane potential, and increased ROS production. Excessive ROS further exacerbates nuclear DNA damage. Mitophagy can mitigate ROS production by clearing damaged mitochondria, thereby reducing oxidative stress and cytotoxicity induced by chemotherapy drugs [41]. It has been reported that PINK1 and LC3 are significantly upregulated in patients with esophageal squamous cell carcinoma, and inhibition of mitophagy can restore chemosensitivity in these patients [42]. Mitophagy can also influence chemoresistance in tumor cells by regulating apoptosis and autophagy pathways [43]. In this study, we found that Gem increased ROS production and decreased mitochondrial membrane potential in BCa cells, and knockdown of TPI1 amplified these effects of Gem. Further experiments, including Western blot and immunofluorescence co-localization, revealed that TPI1 knockdown attenuated Gem-induced mitophagy. Additionally, in chemotherapy-resistant human BCa tissues, high TPI1 expression was positively correlated with enhanced mitophagy. These findings indicate that TPI1 promotes Gem resistance in BCa by enhancing mitophagy.

In summary, our study reveals that TPI1 may serve as a critical switch connecting DNA damage and cellular autophagy. When the chemotherapy drug Gem acts on BCa J82 and UMUC3 cells, TPI1 can directly interact with the autophagy-related protein Beclin-1. This interaction prevents the binding of Bcl-2 to Beclin-1 and inhibits the negative regulatory effect of Bcl-2 on Beclin-1, thereby promoting Gem-induced autophagy in BCa cells. This autophagy process clears damaged mitochondria within the cells, maintaining mitochondrial health, reducing excessive intracellular ROS production, and subsequently inhibiting nuclear DNA damage and apoptosis. These findings highlight TPI1 as a potential therapeutic target for BCa and may provide new insights and strategies for BCa chemotherapy.

### Ethics approval and consent to participate

This study was conducted in strict compliance with international ethical guidelines. The Ethics Committee of the Second Hospital of Lanzhou University approved all human tissue research procedures (Approval No. 2024A-1371) and animal experimental protocols (Approval No. D2024-955). For human participants, written informed consent was obtained prior to study enrollment in accordance with the ethical principles established by the Declaration of Helsinki (1964) and its subsequent amendments. Human tissue procurement followed WHO Guiding Principles for Transplantation. Animal experiments adhered to the WMA Statement on Animal Use in Biomedical Research and EU Directive 2010/63/EU standards for pharmacological research, meeting all requirements for experimental design, animal care, and analysis procedures established by internationally recognized regulatory bodies.

## Supplementary information

supplementary figure and table legends

supplementary table

Original western blots

Figure S1

Figure S2

Figure S3

## Data Availability

The raw data used and/or analyzed during the current study are available from the corresponding author on reasonable request.
