# Supplementary material for: TPI1 enhances gemcitabine resistance in bladder cancer by promoting autophagy through activating Beclin-1
Source: Cell Death Dis. 2025 Dec 22;16(1):923. doi: 10.1038/s41419-025-08368-4 (PMC12748767; doi:10.1038/s41419-025-08368-4)
Supplement: Supplementary file 1 — supplementary figure and table legends [file 41419_2025_8368_MOESM1_ESM.docx]

**Supplementary figure legends**

**Fig. S1** TPI1 expression is positively correlated with drug sensitivity in bladder cancer cells. (A-B) Western blot analysis of endogenous TPI1 protein expression in RT-4, 5637, J82, and UMUC3 cell lines and quantification. (C) Cell viability of the four cell lines was assessed by CCK-8 assay after 24 hours of treatment with Gem. Data are presented as mean ± SD from three independent experiments. P values in (B, C) were calculated using a two-tailed unpaired Student's t-test.

**Fig. S2** TPI1 activates autophagy by regulating the Beclin1 core complex to mediate chemoresistance. (A) Immunofluorescence colocalization analysis of TPI1 and Beclin1 in cells. The Pearson's correlation coefficient indicates a strong colocalization between the two proteins. (B-C) Co-IP analysis of the effect of GEM treatment and TPI1 knockdown on the interaction between endogenous Beclin1 and Bcl-2 in J82 (B) and UMUC3 (C) cells. (D-E) Exogenous Co-IP in HEK293T cells validating the direct effect of TPI1 on the Beclin1-Bcl2 interaction. (F-I) Co-IP analysis of the effects of Gem treatment and TPI1 knockdown on the interactions of Beclin1 with VPS34 (F-G), ATG14L, and UVRAG (H-I) in J82 and UMUC3 cells. (J-K) Western blot analysis of the effects of Gem treatment and TPI1 knockdown on the phosphorylation level of Beclin1 (p-Beclin1) in J82 (J) and UMUC3 (K) cells. Data are presented as mean ± SD from three independent experiments. P values in (B, C, F, G, H, I, J, K) were calculated by one-way ANOVA with Tukey's multiple comparisons test. P values in (D, E) were calculated using a two-tailed unpaired Student's t-test.

**Fig. S3** c-Myc is highly expressed in chemoresistant BCa tissues and cells. (A) Representative IHC staining images and quantitative analysis showing the differential expression of c-Myc in chemosensitive versus chemoresistant BCa tissues. (B) Western blot and quantitative analysis of c-Myc protein expression levels in J82, UMUC3 and their Gemcitabine-resistant counterparts (J82r, UMUC3r). Data are presented as mean ± SD from three independent experiments. P values in (A, B) were calculated using a two-tailed unpaired Student's t-test.

**Graphical Abstract.** Research pattern diagram of TPI1 regulation of Gemcitabine resistance in bladder cancer. (by figdraw)

**Supplementary table legends**

**Table S1.** Sequences of the interference TPI1 are the followings

**Table S2.** Antibodies were used to western blot

**Table S3.** Sequences of Primer for Real-time Polymerase Chain Reaction

**Table S4.** Patient inclusion criteria and pathological information

**Table S5.** Mass spectrometry analysis results for the anti-TPI1 immunoprecipit-ation complex
