# Supplementary material for: TPI1 enhances gemcitabine resistance in bladder cancer by promoting autophagy through activating Beclin-1
Source: Cell Death Dis. 2025 Dec 22;16(1):923. doi: 10.1038/s41419-025-08368-4 (PMC12748767; doi:10.1038/s41419-025-08368-4)
Supplement: Supplementary file 2 — supplementary table [file 41419_2025_8368_MOESM2_ESM.docx]

**Table S1** **Sequences of the interference TPI1 are the followings**

| **ShTPI1 1** |
| --- |
| Top strand: |
| 5’-GATCCGCCGTATCATTTATGGAGGCTCTGTGTTCAAGAGACACAGAGCCTCCATAAATGATACGGTTTTTTG-3’ |
| Bottom strand: |
| 5’-AATTCAAAAAACCGTATCATTTATGGAGGCTCTGTGTCTCTTGAACACAGAGCCTCCATAAATGATACGGCG-3’ |
| **ShTPI1 2** |
| Top strand: |
| 5’-GATCCGTCAAGCCCGAATTCGTGGACATCATTTCAAGAGAATGATGTCCACGAATTCGGGCTTGATTTTTTG-3’ |
| Bottom strand: |
| 5’-AATTCAAAAAATCAAGCCCGAATTCGTGGACATCATTCTCTTGAAATGATGTCCACGAATTCGGGCTTGACG |

**Table S2 Antibodies were used to western blot.**

| **Antibodies** | | |
| --- | --- | --- |
| **REAGENT** | **Catalog and Source** | **Dilution** |
| TPI1 | Cat# 10713-1-AP, Proteintech  Cat# 67515-1-Ig, Proteintech | 1:1000  1:5000 |
| β-actin | Cat# 20536-1-AP, Proteintech  Cat# 60008-1-Ig, Proteintech | 1:4000  1:5000 |
| Myc | Cat# 60003-2-Ig, Proteintech  Cat# R1208-1, HUABIO | 1:2000  1:1000 |
| Flag | Cat# 20543-1-AP, Proteintech  Cat# M1403-2, HUABIO | 1:10000  1:5000 |
| p62 | Cat# 18420-1-AP; Proteintech | 1:5000 |
| LC3 | Cat# 14600-1-AP; Proteintech | 1:1000 |
| Beclin1 | Cat# 11306-1-AP; Proteintech | 1:1000 |
| Bcl-2 | Cat# 12789-1-AP; Proteintech | 1:2000 |
| HA | Cat# 51064-2-AP; Proteintech | 1:5000 |
| Tom20 | Cat# 11802-1-AP; Proteintech | 1:5000 |
| Tim23 | Cat# 11123-1-AP; Proteintech | 1:2000 |
| c-Myc | Cat# 10828-1-AP; Proteintech | 1:2000 |
| VPS34 | Cat# 12452-1-AP; Beyotime | 1:1000 |
| ATG14L | Cat# 19491-1-AP; Proteintech | 1:1000 |
| UVRAG | Cat# 29190-1-AP; Proteintech | 1:1000 |
| p-BECLIN1 | Cat# AP1252; Abclonal | 1:500 |
| PINK1 | Cat# PK05715; Abmart | 1:1000 |
| Parkin | Cat#T56641; Abmart | 1:1000 |
| Alexa Fluor 488-labeled Goat Anti-Rabbit IgG(H+L) | Cat# A0423; Beyotime | 1:200 |
| Alexa Fluor 488-labeled Goat Anti-Mouse IgG(H+L) | Cat# A0428; Beyotime | 1:200 |
| Alexa Fluor 555-labeled Donkey Anti-Rabbit IgG(H+L) | Cat# A0453; Beyotime | 1:200 |
| Alexa Fluor 555-labeled Donkey Anti-Mouse IgG(H+L) | Cat# A0460; Beyotime | 1:200 |
| IRDye 680RD Goat anti-Mouse IgG (H+L) | Cat# 926-68070; LIC | 1:20000 |
| IRDye 800CW Goat anti-Rabbit IgG (H+L) | Cat# 926-32211; LIC | 1:20000 |

**Table S3 Sequences of Primer for Real-time Polymerase Chain Reaction**

| **TPI1** |  |
| --- | --- |
| Forward | 5’- CCCAGGAAGTACACGAGAAG-3’ |
| Reverse | 5’-CAGTCACAGAGCCTCCATAAA-3’ |
| **B-actin** |  |
| Forward | 5’- CACCATTGGCAATGAGCGGTTC-3’ |
| Reverse | 5’- AGGTCTTTGCGGATGTCCACGT-3’ |

**Table S4.** **Patient inclusion criteria and pathological information.**

| Category | Basis of Evaluation | Specific Criteria | |
| --- | --- | --- | --- |
| Gemcitabine-Sensitive | Initial response or recurrence time | - Achieves CR/PR, or at least SD lasting ≥6 months - Recurrence occurs ≥6–12 months after discontinuation/completion of therapy | |
| Gemcitabine-Resistant | Lack of efficacy or early recurrence | - Primary resistance: no initial response (SD→PD) or direct progression - Acquired resistance: initial CR/PR/SD, but recurrence/progression within ≤6 months | |
| Gemcitabine-Sensitive patients | age | sex | pathological stage |
| 1 | 66 | male | TaN0M0 |
| 2 | 70 | female | T1N0M0 |
| 3 | 59 | male | T1N1M0 |
| 4 | 68 | female | T1N1M0 |
| 5 | 62 | female | T2N1M0 |
| 6 | 60 | male | T2N1M0 |
| 7 | 69 | female | T2N1M0 |
| 8 | 59 | male | T2N1M0 |
| 9 | 63 | female | T2N2M0 |
| 10 | 57 | female | T2N2M0 |
| Gemcitabine-Resistant patients | age | sex | pathological stage |
| 1 | 60 | male | T1N0M0 |
| 2 | 62 | male | T1N1M0 |
| 3 | 58 | female | T1N1M0 |
| 4 | 64 | male | T2N0M0 |
| 5 | 64 | female | T2N2M0 |
| 6 | 70 | female | T2N0M0 |
| 7 | 63 | female | T2N0M0 |
| 8 | 58 | female | T2N0M0 |
| 9 | 59 | male | T2N1M0 |
| 10 | 68 | male | T3N2M0 |

Abbreviations: CR: Complete Response; PR: Partial Response; SD: Stable Disease; PD: Progressive Disease.

**Table S5. Mass spectrometry analysis results for the anti-TPI1 immunoprecipit-ation complex.**

| **Accession** | **PG.Coverage** | **PG.Qvalue** | **GeneName** | **ProteinDescription** |
| --- | --- | --- | --- | --- |
| P09382 | 91.10% | 0 | LGALS1 | LEG1_HUMAN Galectin-1 |
| P08670 | 91% | 0 | VIM | VIME_HUMAN Vimentin |
| Q99497 | 89.40% | 0 | PARK7 | PARK7_HUMAN Parkinson disease protein 7 |
| P37802 | 88.90% | 0 | TAGLN2 | TAGL2_HUMAN Transgelin-2 |
| P10599 | 88.60% | 0 | TXN | THIO_HUMAN Thioredoxin |
| P07737 | 87.90% | 0 | PFN1 | PROF1_HUMAN Profilin-1 |
| K7ENI6 | 87.80% | 0 | TMEM256-PLSCR3 | K7ENI6_HUMAN TMEM256-PLSCR3 readthrough (NMD candidate) |
| P04075 | 87.40% | 0 | ALDOA | ALDOA_HUMAN Fructose-bisphosphate aldolase A |
| P60174 | 86.70% | 0 | TPI1 | TPIS_HUMAN Triosephosphate isomerase |
| S4R3I5 | 85.40% | 0 | NDUFA3 | S4R3I5_HUMAN NADH dehydrogenase [ubiquinone] 1 alpha subcomplex subunit 3 |
| P35232 | 85.30% | 0 | PHB1 | PHB1_HUMAN Prohibitin 1 |
| C9J0K6 | 85.20% | 0 | SRI | C9J0K6_HUMAN Sorcin |
| P63241 | 85.10% | 0 | EIF5A | IF5A1_HUMAN Eukaryotic translation initiation factor 5A-1 |
| P04792 | 84.90% | 0 | HSPB1 | HSPB1_HUMAN Heat shock protein beta-1 |
| V9HW62 | 84.80% | 0 | HEL-S-74 | V9HW62_HUMAN Lactoylglutathione lyase |
| Q9BWJ5 | 83.70% | 0 | SF3B5 | SF3B5_HUMAN Splicing factor 3B subunit 5 |
| P23396 | 83.50% | 0 | RPS3 | RS3_HUMAN Small ribosomal subunit protein uS3 |
| P31749 | 82.40% | 0 | AKT1 | AKT_HUMAN alpha serine/threonine-protein kinase |
| E5RHG8 | 82% | 0 | ELOC | E5RHG8_HUMAN Elongin-C (Fragment) |
| P07741 | 81.70% | 0 | APRT | APT_HUMAN Adenine phosphoribosyltransferase |
| Q9BQ67 | 80.90% | 0 | GRWD1 | GRWD1_HUMAN Glutamate-rich WD repeat-containing protein 1 |
| A0A2R8Y5L0 | 80.40% | 0 | TSEN15 | A0A2R8Y5L0_HUMAN tRNA splicing endonuclease subunit 15 |
| P49773 | 80.20% | 0 | HINT1 | HINT1_HUMAN Adenosine 5'-monophosphoramidase HINT1 |
| P49721 | 80.10% | 0 | PSMB2 | PSB2_HUMAN Proteasome subunit beta type-2 |
| P63244 | 80.10% | 0 | RACK1 | RACK1_HUMAN Small ribosomal subunit protein RACK1 |
| P31949 | 80% | 0 | S100A11 | S10AB_HUMAN Protein S100-A11 |
| P30041 | 79.90% | 0 | PRDX6 | PRDX6_HUMAN Peroxiredoxin-6 |
| P04406 | 79.70% | 0 | GAPDH | G3P_HUMAN Glyceraldehyde-3-phosphate dehydrogenase |
| P04080 | 79.60% | 0 | CSTB | CYTB_HUMAN Cystatin-B |
| P63172 | 79.60% | 0 | DYNLT1 | DYLT1_HUMAN Dynein light chain Tctex-type 1 |
| O00244 | 79.40% | 0 | ATOX1 | ATOX1_HUMAN Copper transport protein ATOX1 |
| Q99714 | 79.30% | 0 | HSD17B10 | HCD2_HUMAN 3-hydroxyacyl-CoA dehydrogenase type-2 |
| P35325 | 79.20% | 0 | SPRR2B | SPR2B_HUMAN Small proline-rich protein 2B |
| P50990 | 79.20% | 0 | CCT8 | TCPQ_HUMAN T-complex protein 1 subunit theta |
| P30086 | 79.10% | 0 | PEBP1 | PEBP1_HUMAN Phosphatidylethanolamine-binding protein 1 |
| P23528 | 78.90% | 0 | CFL1 | COF1_HUMAN Cofilin-1 |
| P80723 | 78.90% | 0 | BASP1 | BASP1_HUMAN Brain acid soluble protein 1 |
| P10809 | 78.40% | 0 | HSPD1 | CH60_HUMAN 60 kDa heat shock protein, mitochondrial |
| A0A8I5KSY3 | 78.10% | 0 | CYB5B | A0A8I5KSY3_HUMAN Cytochrome b5 type B |
| P25786 | 77.90% | 0 | PSMA1 | PSA1_HUMAN Proteasome subunit alpha type-1 |
| Q9Y5L4 | 77.90% | 0 | TIMM13 | TIM13_HUMAN Mitochondrial import inner membrane translocase subunit Tim13 |
| P51149 | 77.80% | 0 | RAB7A | RAB7A_HUMAN Ras-related protein Rab-7a |
| Q9UI30 | 77.60% | 0 | TRMT112 | TR112_HUMAN Multifunctional methyltransferase subunit TRM112-like protein |
| P00558 | 77.50% | 0 | PGK1 | PGK1_HUMAN Phosphoglycerate kinase 1 |
| Q15181 | 77.50% | 0 | PPA1 | IPYR_HUMAN Inorganic pyrophosphatase |
| P05387 | 77.40% | 0 | RPLP2 | RLA2_HUMAN Large ribosomal subunit protein P2 |
| P16152 | 77.30% | 0 | CBR1 | CBR1_HUMAN Carbonyl reductase [NADPH] 1 |
| P12004 | 77% | 0 | PCNA | PCNA_HUMAN Proliferating cell nuclear antigen |
| P00338 | 76.80% | 0 | LDHA | LDHA_HUMAN L-lactate dehydrogenase A chain |
| P17987 | 76.80% | 0 | TCP1 | TCPA_HUMAN T-complex protein 1 subunit alpha |
| Q9NS69 | 76.80% | 0 | TOMM22 | TOM22_HUMAN Mitochondrial import receptor subunit TOM22 homolog |
| Q99471 | 76.60% | 0 | PFDN5 | PFD5_HUMAN Prefoldin subunit 5 |
| A0A024R5Z7 | 76.40% | 0 | ANXA2 | A0A024R5Z7_HUMAN Annexin |
| V9HW65 | 76.40% | 0 | HEL-S-270 | V9HW65_HUMAN Annexin |
| O75947 | 76.40% | 0 | ATP5PD | ATP5H_HUMAN ATP synthase subunit d, mitochondrial |
| D6RAN8 | 76.30% | 0 | MRPL27 | D6RAN8_HUMAN Mitochondrial ribosomal protein L27 |
| Q00987 | 76.20% | 0 | MDM2 | MDM2_HUMAN Double minute 2 protein (Hdm2) |
| Q15102 | 76.20% | 0 | PAFAH1B3 | PA1B3_HUMAN Platelet-activating factor acetylhydrolase IB subunit alpha1 |
| D3DPU2 | 76% | 0 | CAP1 | D3DPU2_HUMAN Adenylyl cyclase-associated protein |
| P21796 | 76% | 0 | VDAC1 | VDAC1_HUMAN Non-selective voltage-gated ion channel VDAC1 |
| Q9Y3F4 | 76% | 0 | STRAP | STRAP_HUMAN Serine-threonine kinase receptor-associated protein |
| P54920 | 75.90% | 0 | NAPA | SNAA_HUMAN Alpha-soluble NSF attachment protein |
| P63104 | 75.90% | 0 | YWHAZ | 1433Z_HUMAN 14-3-3 protein zeta/delta |
| P78371 | 75.90% | 0 | CCT2 | TCPB_HUMAN T-complex protein 1 subunit beta |
| Q9BYN0 | 75.90% | 0 | SRXN1 | SRXN1_HUMAN Sulfiredoxin-1 |
| Q9UBE0 | 75.70% | 0 | SAE1 | SAE1_HUMAN SUMO-activating enzyme subunit 1 |
| P14868 | 75.40% | 0 | DARS1 | SYDC_HUMAN Aspartate--tRNA ligase, cytoplasmic |
| Q14019 | 75.40% | 0 | COTL1 | COTL1_HUMAN Coactosin-like protein |
| Q9NX24 | 75.20% | 0 | NHP2 | NHP2_HUMAN H/ACA ribonucleoprotein complex subunit 2 |
| Q9NX55 | 75.20% | 0 | HYPK | HYPK_HUMAN Huntingtin-interacting protein K |
| Q9H0U4 | 75.10% | 0 | RAB1B | RAB1B_HUMAN Ras-related protein Rab-1B |
| A0A8I5KUS5 | 75% | 0 | ELOB | A0A8I5KUS5_HUMAN Elongin B |
| Q96A72 | 75% | 0 | MAGOHB | MGN2_HUMAN Protein mago nashi homolog 2 |
| P61970 | 74.80% | 0 | NUTF2 | NTF2_HUMAN Nuclear transport factor 2 |
| P05386 | 74.60% | 0 | RPLP1 | RLA1_HUMAN Large ribosomal subunit protein P1 |
| A0A7P0TAW3 | 74.50% | 0 | VCP | A0A7P0TAW3_HUMAN Transitional endoplasmic reticulum ATPase |
| Q6IAA8 | 74.50% | 0 | LAMTOR1 | LTOR1_HUMAN Ragulator complex protein LAMTOR1 |
| P14854 | 74.40% | 0 | COX6B1 | CX6B1_HUMAN Cytochrome c oxidase subunit 6B1 |
| A0A087WVQ9 | 74.30% | 0 | EEF1A1 | A0A087WVQ9_HUMAN Elongation factor 1-alpha |
| P11413 | 74.20% | 0 | G6PD | G6PD_HUMAN Glucose-6-phosphate 1-dehydrogenase |
| O00429 | 74.20% | 0 | DNM1L | DNM1L_HUMAN Dynamin-1-like protein |
| O60493 | 74.10% | 0 | SNX3 | SNX3_HUMAN Sorting nexin-3 |
| P50583 | 74.10% | 0 | NUDT2 | AP4A_HUMAN Bis(5'-nucleosyl)-tetraphosphatase [asymmetrical] |
| O95433 | 74% | 0 | AHSA1 | AHSA1_HUMAN Activator of 90 kDa heat shock protein ATPase homolog 1 |
| O95777 | 74% | 0 | LSM8 | LSM8_HUMAN U6 snRNA-associated Sm-like protein LSm8 |
| P14618 | 74% | 0 | PKM | KPYM_HUMAN Pyruvate kinase PKM |
| P62249 | 74% | 0 | RPS16 | RS16_HUMAN Small ribosomal subunit protein uS9 |
| P60981 | 73.90% | 0 | DSTN | DEST_HUMAN Destrin |
| Q06830 | 73.90% | 0 | PRDX1 | PRDX1_HUMAN Peroxiredoxin-1 |
| V9HW35 | 73.50% | 0 | HEL-S-55 | V9HW35_HUMAN Peroxiredoxin-5 |
| Q99832 | 73.50% | 0 | CCT7 | TCPH_HUMAN T-complex protein 1 subunit eta |
| P46926 | 73.40% | 0 | GNPDA1 | GNPI1_HUMAN Glucosamine-6-phosphate isomerase 1 |
| P21266 | 73.30% | 0 | GSTM3 | GSTM3_HUMAN Glutathione S-transferase Mu 3 |
| P31939 | 73.30% | 0 | ATIC | PUR9_HUMAN Bifunctional purine biosynthesis protein ATIC |
| P08758 | 73.10% | 0 | ANXA5 | ANXA5_HUMAN Annexin A5 |
| P61106 | 73% | 0 | RAB14 | RAB14_HUMAN Ras-related protein Rab-14 |
| Q13637 | 72.90% | 0 | RAB32 | RAB32_HUMAN Ras-related protein Rab-32 |
| Q02790 | 72.80% | 0 | FKBP4 | FKBP4_HUMAN Peptidyl-prolyl cis-trans isomerase FKBP4 |
| P07437 | 72.70% | 0 | TUBB | TBB5_HUMAN Tubulin beta chain |
| P06493 | 72.70% | 0 | CDK1 | CDK1_HUMAN Cyclin-dependent kinase 1 |
| O75506 | 72.40% | 0 | HSBP1 | HSBP1_HUMAN Heat shock factor-binding protein 1 |
| O43447 | 72.30% | 0 | PPIH | PPIH_HUMAN Peptidyl-prolyl cis-trans isomerase H |
| P13639 | 72.30% | 0 | EEF2 | EF2_HUMAN Elongation factor 2 |
| Q9H3K6 | 72.10% | 0 | BOLA2 | BOLA2_HUMAN BolA-like protein 2 |
| Q15691 | 72% | 0 | MAPRE1 | MARE1_HUMAN Microtubule-associated protein RP/EB family member 1 |
| P08708 | 71.90% | 0 | RPS17 | RS17_HUMAN Small ribosomal subunit protein eS17 |
| K7EM18 | 71.90% | 0 | EIF1 | K7EM18_HUMAN Eukaryotic translation initiation factor 1 |
| Q01469 | 71.90% | 0 | FABP5 | FABP5_HUMAN Fatty acid-binding protein 5 |
| Q9NZL4 | 71.90% | 0 | HSPBP1 | HPBP1_HUMAN Hsp70-binding protein 1 |
| Q9UNQ2 | 71.90% | 0 | DIMT1 | DIM1_HUMAN Dimethyladenosine transferase |
| Q08J23 | 71.70% | 0 | NSUN2 | NSUN2_HUMAN RNA cytosine C(5)-methyltransferase NSUN2 |
| Q92979 | 71.70% | 0 | EMG1 | NEP1_HUMAN Ribosomal RNA small subunit methyltransferase NEP1 |
| Q9NR31 | 71.70% | 0 | SAR1A | SAR1A_HUMAN Small COPII coat GTPase SAR1A |
| P55263 | 71.50% | 0 | ADK | ADK_HUMAN Adenosine kinase |
| P19623 | 71.50% | 0 | SRM | SPEE_HUMAN Spermidine synthase |
| O00299 | 71.40% | 0 | CLIC1 | CLIC1_HUMAN Chloride intracellular channel protein 1 |
| P62942 | 71.30% | 0 | FKBP1A | FKB1A_HUMAN Peptidyl-prolyl cis-trans isomerase FKBP1A |
| P25398 | 71.20% | 0 | RPS12 | RS12_HUMAN Small ribosomal subunit protein eS12 |
| Q32Q12 | 71.20% | 0 | NME1-NME2 | Q32Q12_HUMAN Nucleoside diphosphate kinase |
| Q9GZL7 | 71.20% | 0 | WDR12 | WDR12_HUMAN Ribosome biogenesis protein WDR12 |
| A0A3S6H812 | 71% | 0 | PRMT1 | A0A3S6H812_HUMAN type I protein arginine methyltransferase |
| P47756 | 71% | 0 | CAPZB | CAPZB_HUMAN F-actin-capping protein subunit beta |
| F8W1R7 | 71% | 0 | MYL6 | F8W1R7_HUMAN Myosin light chain 6 |
| P06733 | 71% | 0 | ENO1 | ENOA_HUMAN Alpha-enolase |
| P25789 | 70.90% | 0 | PSMA4 | PSA4_HUMAN Proteasome subunit alpha type-4 |
| P49368 | 70.80% | 0 | CCT3 | TCPG_HUMAN T-complex protein 1 subunit gamma |
| P49411 | 70.80% | 0 | TUFM | EFTU_HUMAN Elongation factor Tu, mitochondrial |
| P06576 | 70.70% | 0 | ATP5F1B | ATPB_HUMAN ATP synthase subunit beta, mitochondrial |
| P50395 | 70.60% | 0 | GDI2 | GDIB_HUMAN Rab GDP dissociation inhibitor beta |
| A0A024R324 | 70.50% | 0 | RHOA | A0A024R324_HUMAN Transforming protein RhoA |
| P67809 | 70.40% | 0 | YBX1 | YBOX1_HUMAN Y-box-binding protein 1 |
| P62937 | 70.30% | 0 | PPIA | PPIA_HUMAN Peptidyl-prolyl cis-trans isomerase A |
| Q9UJV9 | 70.30% | 0 | DDX41 | DDX41_HUMAN Probable ATP-dependent RNA helicase DDX41 |
| O00151 | 70.20% | 0 | PDLIM1 | PDLI1_HUMAN PDZ and LIM domain protein 1 |
| Q9UKY7 | 70.20% | 0 | CDV3 | CDV3_HUMAN Protein CDV3 homolog |
| P62829 | 70% | 0 | RPL23 | RL23_HUMAN Large ribosomal subunit protein uL14 |
| Q9Y6G9 | 70% | 0 | DYNC1LI1 | DC1L1_HUMAN Cytoplasmic dynein 1 light intermediate chain 1 |
| P10768 | 69.90% | 0 | ESD | ESTD_HUMAN S-formylglutathione hydrolase |
| P05198 | 69.80% | 0 | EIF2S1 | IF2A_HUMAN Eukaryotic translation initiation factor 2 subunit 1 |
| P32969 | 69.80% | 0 | RPL9 | RL9_HUMAN Large ribosomal subunit protein uL6 |
| P60842 | 69.70% | 0 | EIF4A1 | IF4A1_HUMAN Eukaryotic initiation factor 4A-I |
| P04083 | 69.70% | 0 | ANXA1 | ANXA1_HUMAN Annexin A1 |
| C9JX88 | 69.50% | 0 | PSMC2 | C9JX88_HUMAN 26S proteasome regulatory subunit 7 |
| Q9UQ80 | 69.50% | 0 | PA2G4 | PA2G4_HUMAN Proliferation-associated protein 2G4 |
| P13489 | 69.40% | 0 | RNH1 | RINI_HUMAN Ribonuclease inhibitor |
| P62140 | 69.40% | 0 | PPP1CB | PP1B_HUMAN Serine/threonine-protein phosphatase PP1-beta catalytic subunit |
| Q9NQR4 | 69.20% | 0 | NIT2 | NIT2_HUMAN Omega-amidase NIT2 |
| Q9Y696 | 69.20% | 0 | CLIC4 | CLIC4_HUMAN Chloride intracellular channel protein 4 |
| O75223 | 69.10% | 0 | GGCT | GGCT_HUMAN Gamma-glutamylcyclotransferase |
| P02533 | 69.10% | 0 | KRT14 | K1C14_HUMAN Keratin, type I cytoskeletal 14 |
| O75438 | 69% | 0 | NDUFB1 | NDUB1_HUMAN NADH dehydrogenase [ubiquinone] 1 beta subcomplex subunit 1 |
| Q14457 | 68.90% | 0 | Beclin1 | Beclin1_HUMAN Beclin1 |
| Q15738 | 68.90% | 0 | NSDHL | NSDHL_HUMAN Sterol-4-alpha-carboxylate 3-dehydrogenase, decarboxylating |
| P78346 | 68.70% | 0 | RPP30 | RPP30_HUMAN Ribonuclease P protein subunit p30 |
| Q8N4H5 | 68.60% | 0 | TOMM5 | TOM5_HUMAN Mitochondrial import receptor subunit TOM5 homolog |
| O00567 | 68.50% | 0 | NOP56 | NOP56_HUMAN Nucleolar protein 56 |
| P18669 | 68.50% | 0 | PGAM1 | PGAM1_HUMAN Phosphoglycerate mutase 1 |
| Q9Y5J7 | 68.50% | 0 | TIMM9 | TIM9_HUMAN Mitochondrial import inner membrane translocase subunit Tim9 |
| P12814 | 68.40% | 0 | ACTN1 | ACTN1_HUMAN Alpha-actinin-1 |
| P62701 | 68.40% | 0 | RPS4X | RS4X_HUMAN Small ribosomal subunit protein eS4, X isoform |
| C9JLU1 | 68.20% | 0 | POLR2H | C9JLU1_HUMAN RNA polymerase II, I and III subunit H (Fragment) |
| P61960 | 68.20% | 0 | UFM1 | UFM1_HUMAN Ubiquitin-fold modifier 1 |
| P07203 | 68% | 0 | GPX1 | GPX1_HUMAN Glutathione peroxidase 1 |
| Q15149 | 68% | 0 | PLEC | PLEC_HUMAN Plectin |
| P35244 | 67.80% | 0 | RPA3 | RFA3_HUMAN Replication protein A 14 kDa subunit |
| P40926 | 67.80% | 0 | MDH2 | MDHM_HUMAN Malate dehydrogenase, mitochondrial |
| P52272 | 67.80% | 0 | HNRNPM | HNRPM_HUMAN Heterogeneous nuclear ribonucleoprotein M |
| P62316 | 67.80% | 0 | SNRPD2 | SMD2_HUMAN Small nuclear ribonucleoprotein Sm D2 |
| P41227 | 67.70% | 0 | NAA10 | NAA10_HUMAN N-alpha-acetyltransferase 10 |
| Q09666 | 67.70% | 0 | AHNAK | AHNK_HUMAN Neuroblast differentiation-associated protein AHNAK |
| P61604 | 67.60% | 0 | HSPE1 | CH10_HUMAN 10 kDa heat shock protein, mitochondrial |
| O43809 | 67.40% | 0 | NUDT21 | CPSF5_HUMAN Cleavage and polyadenylation specificity factor subunit 5 |
| Q9Y333 | 67.40% | 0 | LSM2 | LSM2_HUMAN U6 snRNA-associated Sm-like protein LSm2 |
| P48643 | 67.30% | 0 | CCT5 | TCPE_HUMAN T-complex protein 1 subunit epsilon |
| Q6PJ77 | 67.30% | 0 | BTF3L4 | Q6PJ77_HUMAN Transcription factor BTF3 (Fragment) |
| Q8WXX5 | 67.30% | 0 | DNAJC9 | DNJC9_HUMAN DnaJ homolog subfamily C member 9 |
| P68363 | 67.20% | 0 | TUBA1B | TBA1B_HUMAN Tubulin alpha-1B chain |
| P02795 | 67.20% | 0 | MT2A | MT2_HUMAN Metallothionein-2 |
| P15880 | 67.20% | 0 | RPS2 | RS2_HUMAN Small ribosomal subunit protein uS5 |
| P43686 | 67.20% | 0 | PSMC4 | PRS6B_HUMAN 26S proteasome regulatory subunit 6B |
| P11177 | 67.10% | 0 | PDHB | ODPB_HUMAN Pyruvate dehydrogenase E1 component subunit beta, mitochondrial |
| P54136 | 67.10% | 0 | RARS1 | SYRC_HUMAN Arginine--tRNA ligase, cytoplasmic |
| E9PM69 | 67% | 0 | PSMC3 | E9PM69_HUMAN Proteasome 26S subunit, ATPase 3 |
| P61019 | 67% | 0 | RAB2A | RAB2A_HUMAN Ras-related protein Rab-2A |
| P62081 | 67% | 0 | RPS7 | RS7_HUMAN Small ribosomal subunit protein eS7 |
| P62888 | 67% | 0 | RPL30 | RL30_HUMAN Large ribosomal subunit protein eL30 |
| Q9P015 | 66.90% | 0 | MRPL15 | RM15_HUMAN Large ribosomal subunit protein uL15m |
| Q9BYG3 | 66.90% | 0 | NIFK | MK67I_HUMAN MKI67 FHA domain-interacting nucleolar phosphoprotein |
| P14550 | 66.80% | 0 | AKR1A1 | AK1A1_HUMAN Aldo-keto reductase family 1 member A1 |
| A0A804HI79 | 66.70% | 0 | RP9 | A0A804HI79_HUMAN RP9 pre-mRNA splicing factor |
| G3V5Z7 | 66.70% | 0 | PSMA6 | G3V5Z7_HUMAN Proteasome subunit alpha type |
| P08133 | 66.70% | 0 | ANXA6 | ANXA6_HUMAN Annexin A6 |
| P22532 | 66.70% | 0 | SPRR2D | SPR2D_HUMAN Small proline-rich protein 2D |
| P24752 | 66.70% | 0 | ACAT1 | THIL_HUMAN Acetyl-CoA acetyltransferase, mitochondrial |
| P55084 | 66.70% | 0 | HADHB | ECHB_HUMAN Trifunctional enzyme subunit beta, mitochondrial |
| Q9UBI6 | 66.70% | 0 | GNG12 | GBG12_HUMAN Guanine nucleotide-binding protein G(I)/G(S)/G(O) subunit gamma-12 |
| P17174 | 66.60% | 0 | GOT1 | AATC_HUMAN Aspartate aminotransferase, cytoplasmic |
| K7ELC2 | 66.40% | 0 | RPS15 | K7ELC2_HUMAN Small ribosomal subunit protein uS19 |
| Q9NT62 | 66.40% | 0 | ATG3 | ATG3_HUMAN Ubiquitin-like-conjugating enzyme ATG3 |
| P48739 | 66.40% | 0 | PITPNB | PIPNB_HUMAN Phosphatidylinositol transfer protein beta isoform |
| Q16718 | 66.40% | 0 | NDUFA5 | NDUA5_HUMAN NADH dehydrogenase [ubiquinone] 1 alpha subcomplex subunit 5 |
| Q9Y2Z0 | 66.30% | 0 | SUGT1 | SGT1_HUMAN Protein SGT1 homolog |
| P29692 | 66.20% | 0 | EEF1D | EF1D_HUMAN Elongation factor 1-delta |
| P55060 | 66.20% | 0 | CSE1L | XPO2_HUMAN Exportin-2 |
| Q9UNM6 | 66.20% | 0 | PSMD13 | PSD13_HUMAN 26S proteasome non-ATPase regulatory subunit 13 |
| Q9Y3B7 | 66.10% | 0 | MRPL11 | RM11_HUMAN Large ribosomal subunit protein uL11m |
| P05783 | 66% | 0 | KRT18 | K1C18_HUMAN Keratin, type I cytoskeletal 18 |
| P23919 | 66% | 0 | DTYMK | KTHY_HUMAN Thymidylate kinase |
| P46781 | 66% | 0 | RPS9 | RS9_HUMAN Small ribosomal subunit protein uS4 |
| Q9UHD1 | 66% | 0 | CHORDC1 | CHRD1_HUMAN Cysteine and histidine-rich domain-containing protein 1 |
| A0A804HKI2 | 65.90% | 0 | LRPPRC | A0A804HKI2_HUMAN Leucine rich pentatricopeptide repeat containing |
| O95352 | 65.90% | 0 | ATG7 | ATG7_HUMAN Ubiquitin-like modifier-activating enzyme ATG7 |
| O60664 | 65.90% | 0 | PLIN3 | PLIN3_HUMAN Perilipin-3 |
| P61247 | 65.90% | 0 | RPS3A | RS3A_HUMAN Small ribosomal subunit protein eS1 |
| Q9Y230 | 65.90% | 0 | RUVBL2 | RUVB2_HUMAN RuvB-like 2 |
| Q15366 | 65.80% | 0 | PCBP2 | PCBP2_HUMAN Poly(rC)-binding protein 2 |
| A0A8V8TNZ5 | 65.80% | 0 | ARF1 | A0A8V8TNZ5_HUMAN ADP-ribosylation factor (Fragment) |
| A0A7I2V2G2 | 65.70% | 0 | HSPA9 | A0A7I2V2G2_HUMAN Stress-70 protein, mitochondrial |
| Q15365 | 65.70% | 0 | PCBP1 | PCBP1_HUMAN Poly(rC)-binding protein 1 |
| Q9H4M9 | 65.70% | 0 | EHD1 | EHD1_HUMAN EH domain-containing protein 1 |
| Q99623 | 65.60% | 0 | PHB2 | PHB2_HUMAN Prohibitin-2 |
| F8W1A4 | 65.50% | 0 | AK2 | F8W1A4_HUMAN Adenylate kinase 2, mitochondrial |
| Q12905 | 65.40% | 0 | ILF2 | ILF2_HUMAN Interleukin enhancer-binding factor 2 |
| O15144 | 65.30% | 0 | ARPC2 | ARPC2_HUMAN Actin-related protein 2/3 complex subunit 2 |
| Q8NEB9 | 65.30% | 0 | VPS34 | VPS34_HUMAN Phosphatidylinositol 3-kinase catalytic subunit type 3 |
| P51148 | 65.30% | 0 | RAB5C | RAB5C_HUMAN Ras-related protein Rab-5C |
| P67936 | 65.30% | 0 | TPM4 | TPM4_HUMAN Tropomyosin alpha-4 chain |
| P02768 | 65.20% | 0 | ALB | ALBU_HUMAN Albumin |
| P22061 | 65.20% | 0 | PCMT1 | PIMT_HUMAN Protein-L-isoaspartate(D-aspartate) O-methyltransferase |
| Q9Y4P1 | 65.20% | 0 | ATG4B | ATG4B_HUMAN Cysteine protease ATG4B |
| Q7Z4H3 | 65.20% | 0 | HDDC2 | HDDC2_HUMAN 5'-deoxynucleotidase HDDC2 |
| P08559 | 65.10% | 0 | PDHA1 | ODPA_HUMAN Pyruvate dehydrogenase E1 component subunit alpha, somatic form, mitochondrial |
| Q9UBQ5 | 65.10% | 0 | EIF3K | EIF3K_HUMAN Eukaryotic translation initiation factor 3 subunit K |
