# Supplementary material for: TPI1 enhances gemcitabine resistance in bladder cancer by promoting autophagy through activating Beclin-1
Source: Cell Death Dis. 2025 Dec 22;16(1):923. doi: 10.1038/s41419-025-08368-4 (PMC12748767; doi:10.1038/s41419-025-08368-4)
Supplement: Supplementary file 3 — Original western blots [file 41419_2025_8368_MOESM3_ESM.pdf]

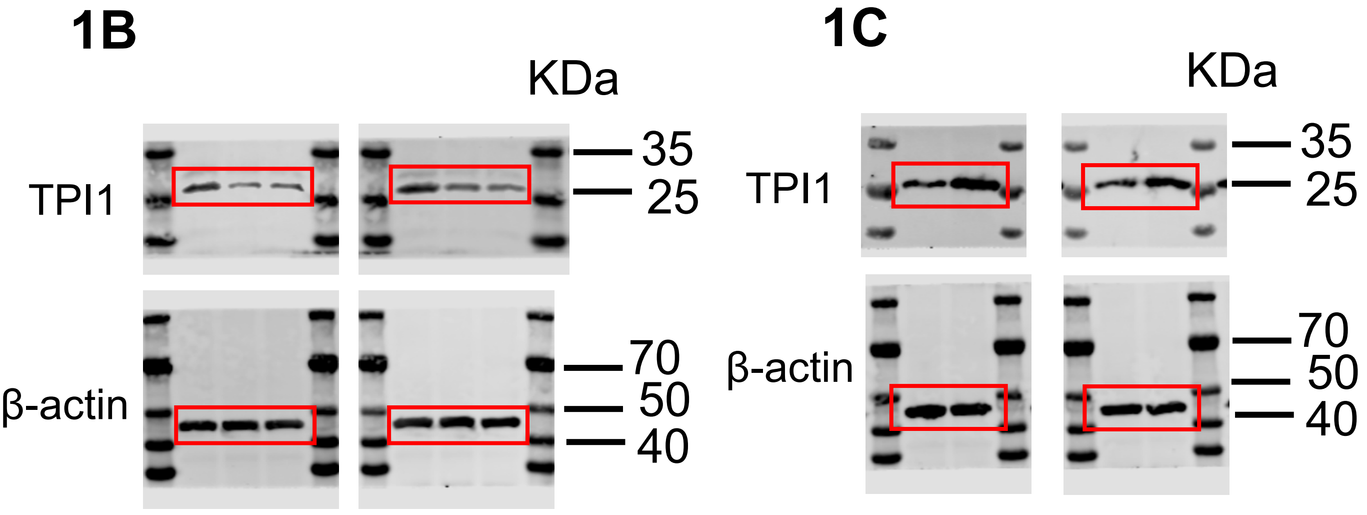

Fig.1 Unprocessed immunoblots for indicated Figures panels.

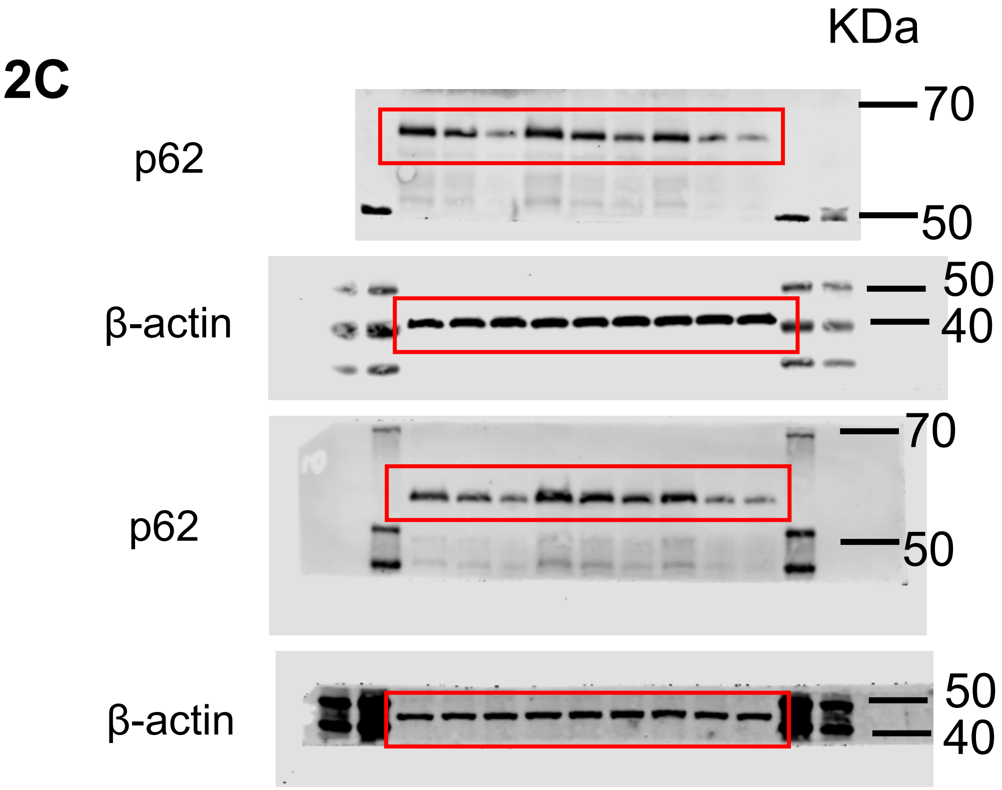

Fig.2 Unprocessed immunoblots for indicated Figures panels.

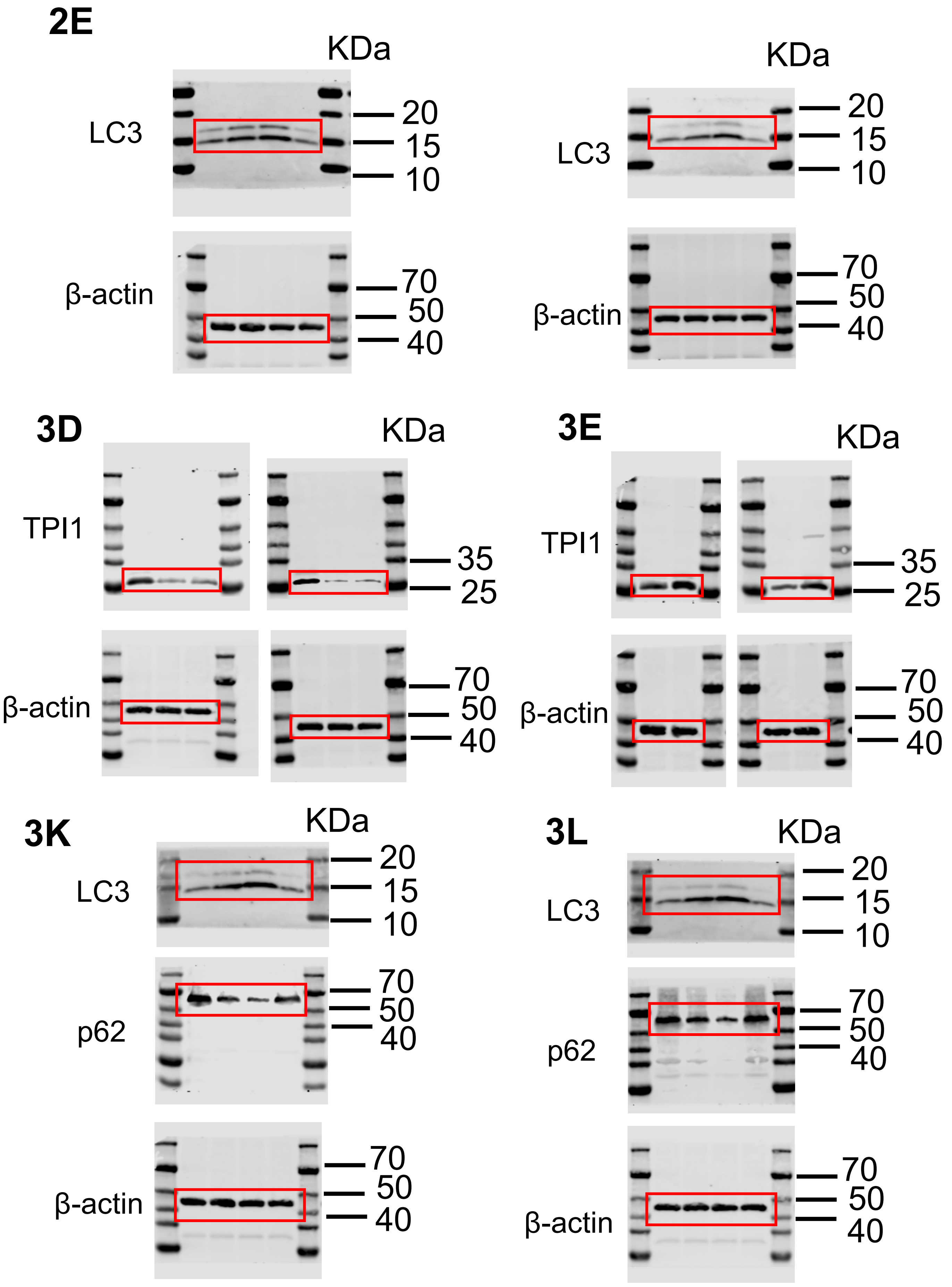

Fig.3 Unprocessed immunoblots for indicated Figures panels.

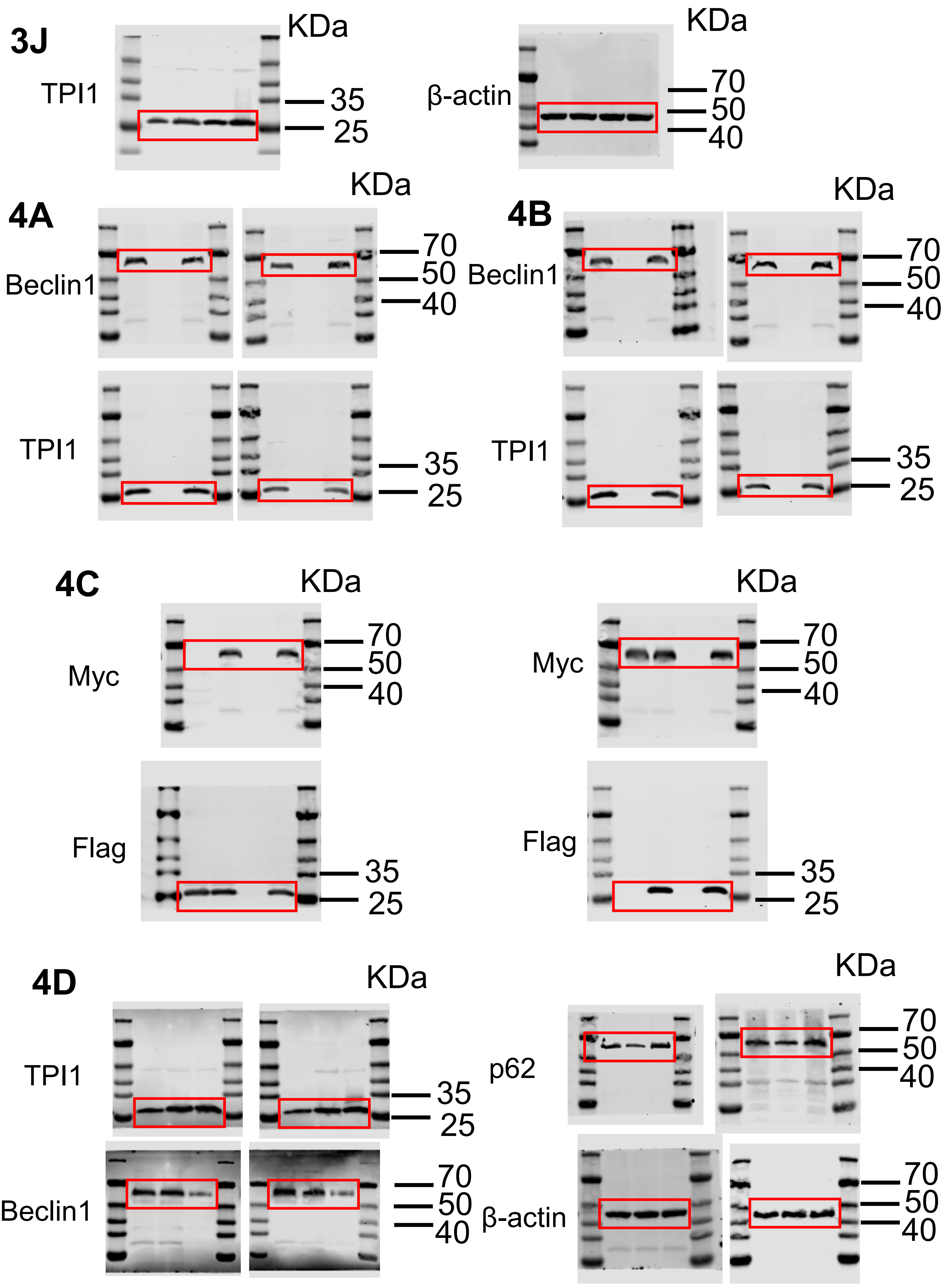

Fig.4 Unprocessed immunoblots for indicated Figures panels.

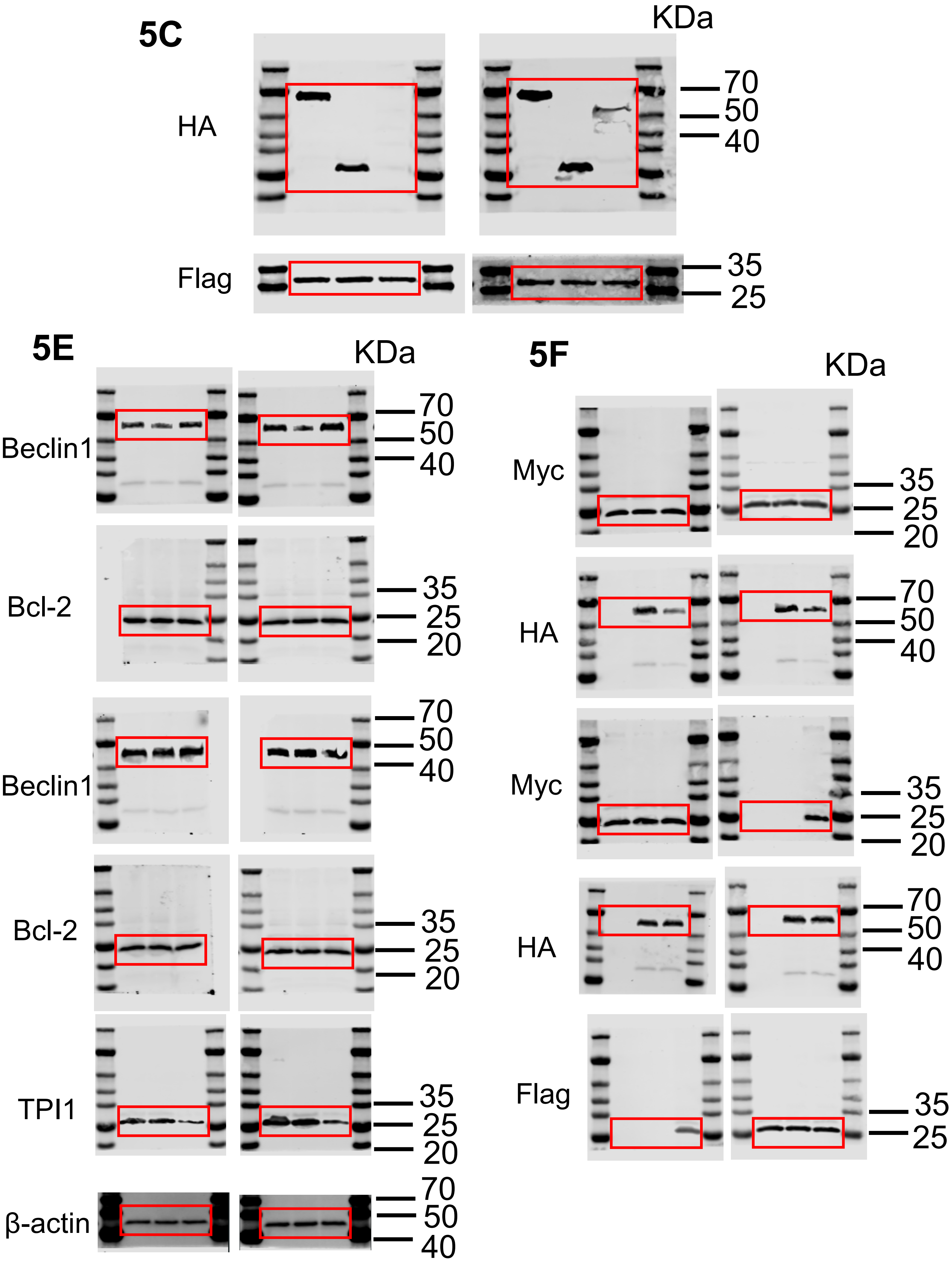

Fig.5 Unprocessed immunoblots for indicated Figures panels.

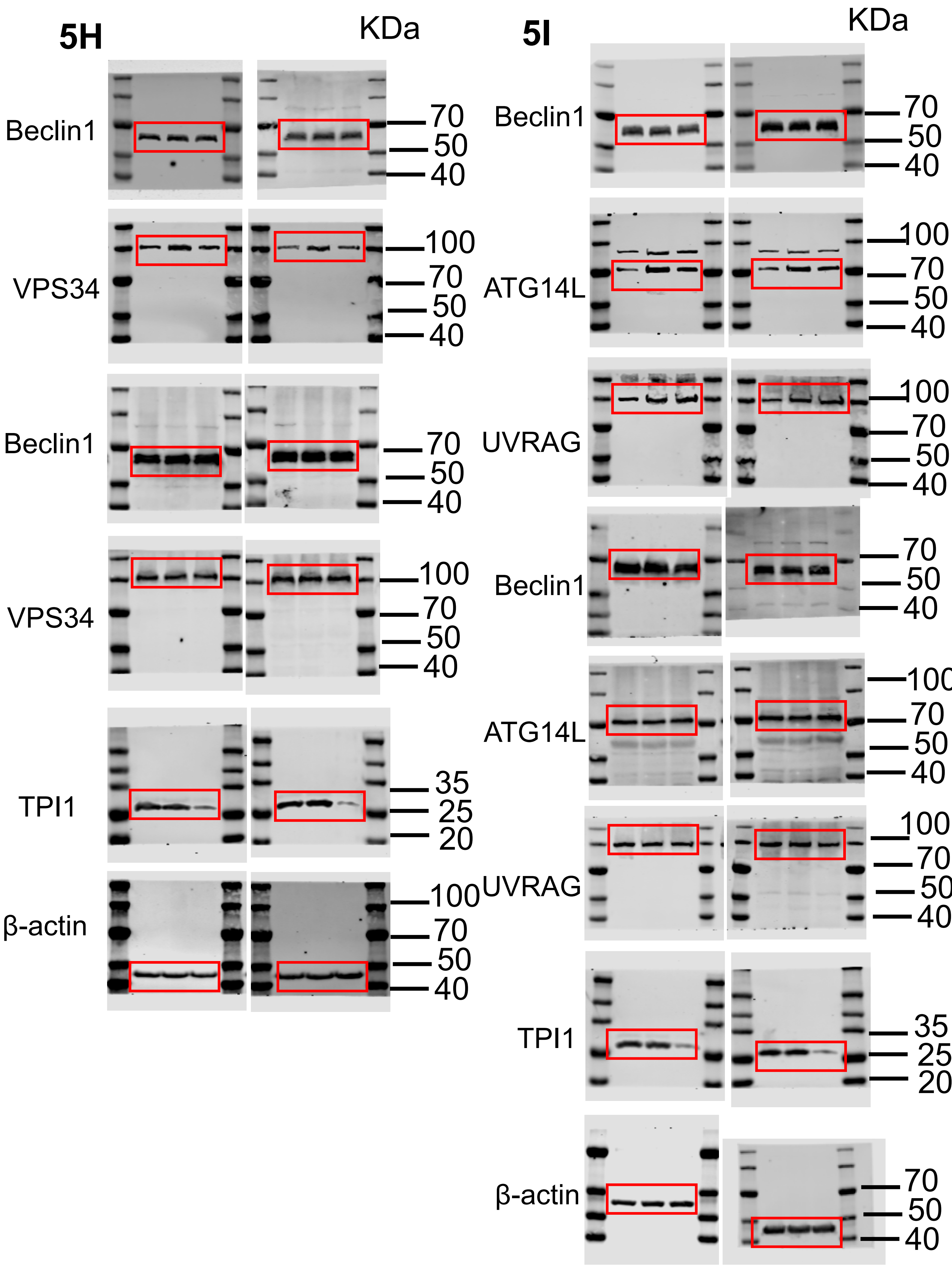

Fig.5 Unprocessed immunoblots for indicated Figures panels.

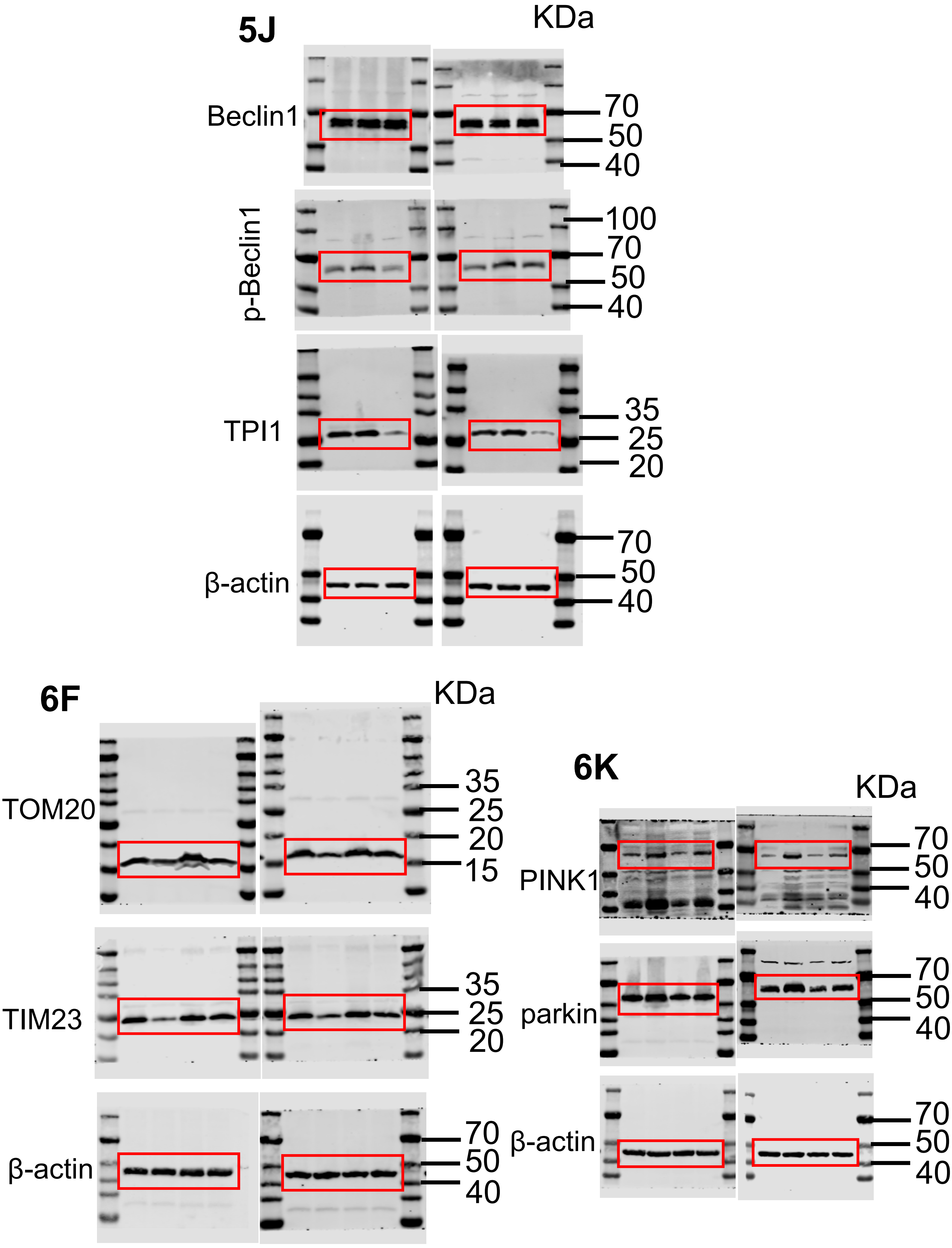

Fig.6 Unprocessed immunoblots for indicated Figures panels.

7A

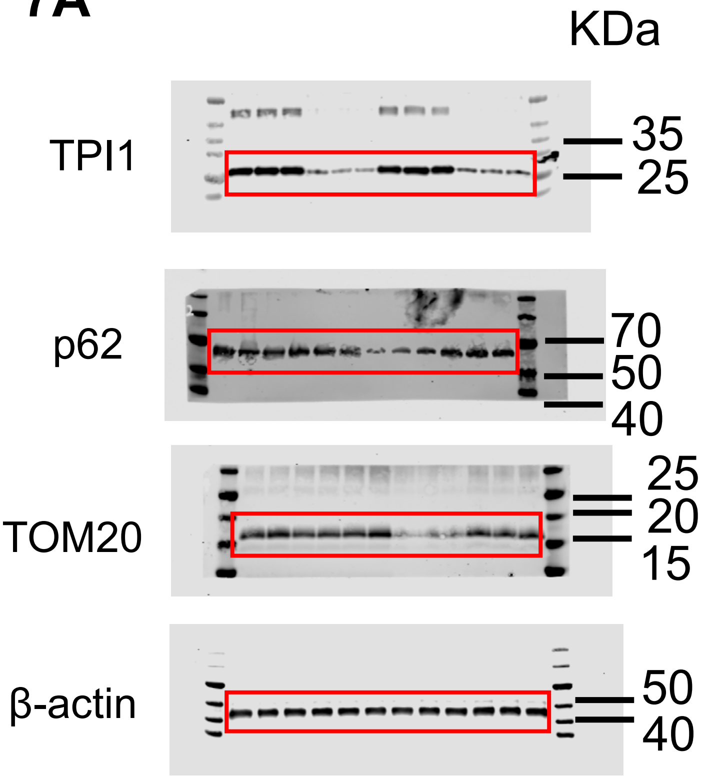

7C

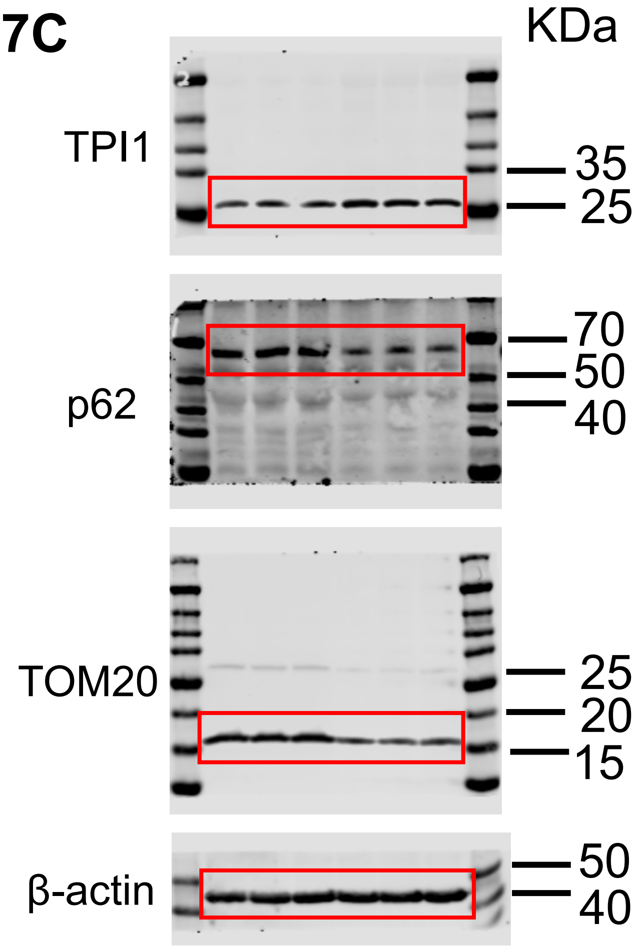

Fig.7 Unprocessed immunoblots for indicated Figures panels.

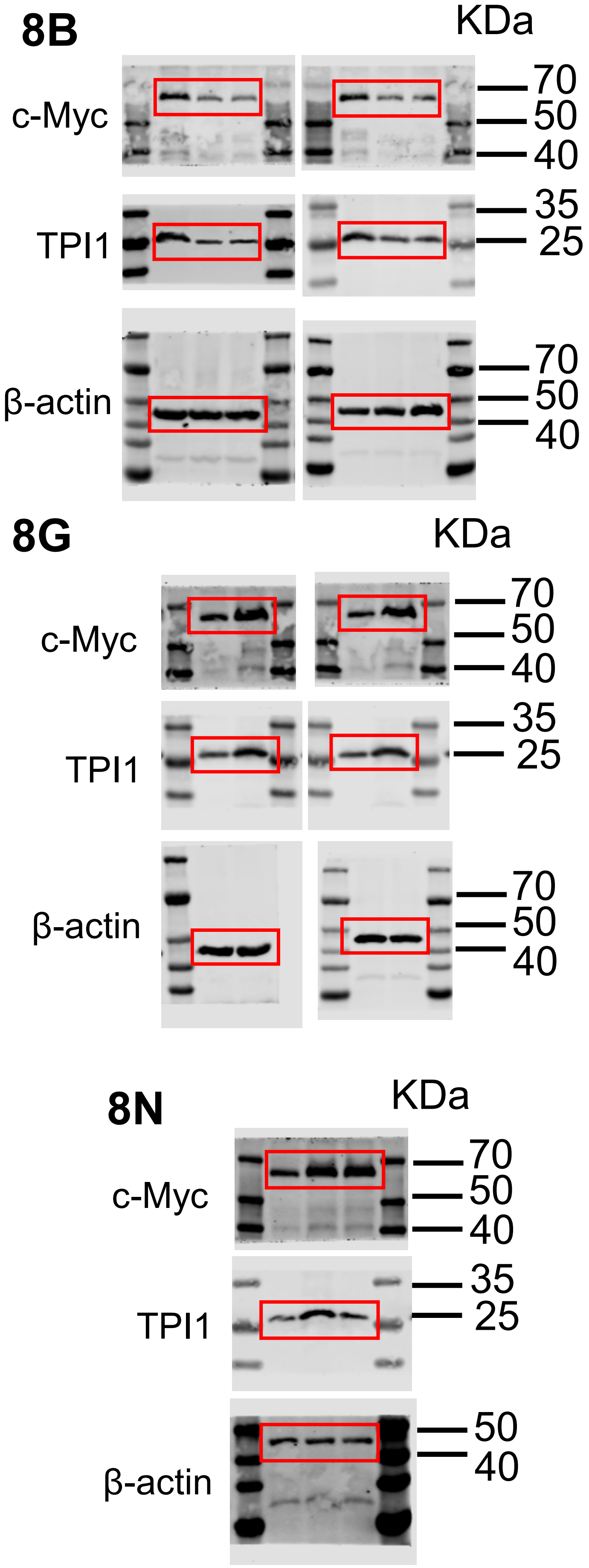

Fig.8 Unprocessed immunoblots for indicated Figures panels.

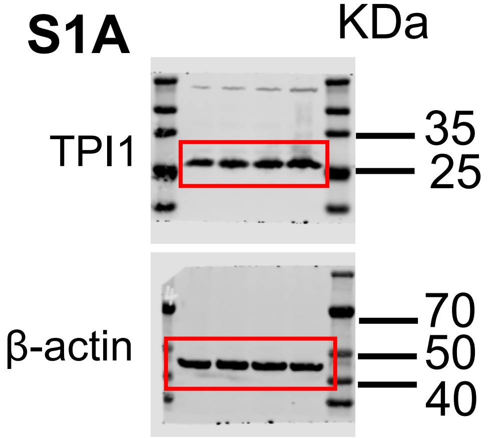

Fig.S1 Unprocessed immunoblots for indicated Figures panels.

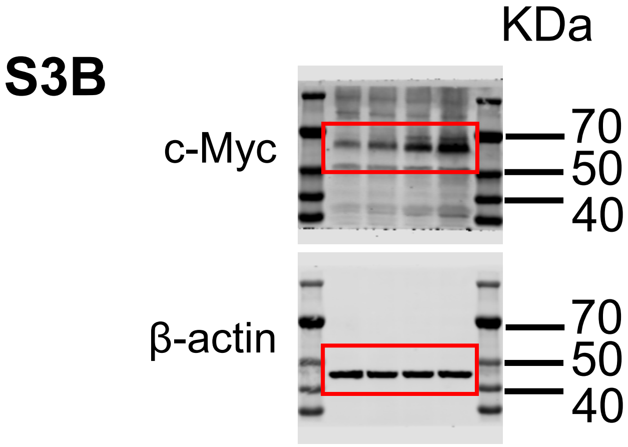

Fig.S3 Unprocessed immunoblots for indicated Figures panels.
