# Supplementary figures and images for: TPI1 enhances gemcitabine resistance in bladder cancer by promoting autophagy through activating Beclin-1
Source: Cell Death Dis. 2025 Dec 22;16(1):923. doi: 10.1038/s41419-025-08368-4 (PMC12748767; doi:10.1038/s41419-025-08368-4)

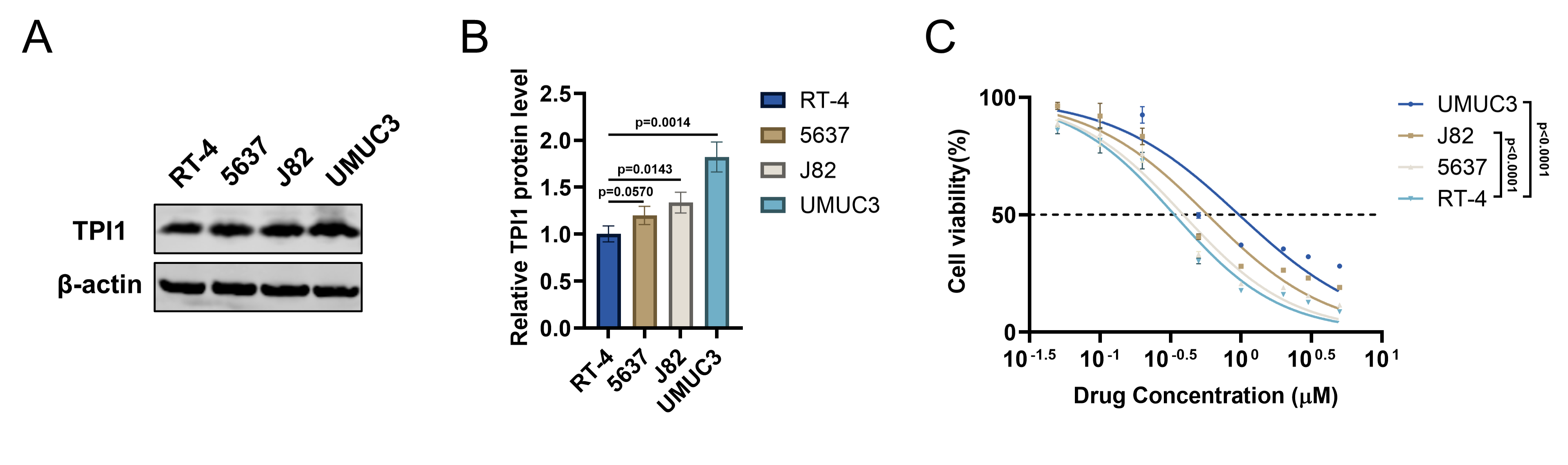

Supplement: Supplementary file 4 — Figure S1 [file 41419_2025_8368_MOESM4_ESM.tif]

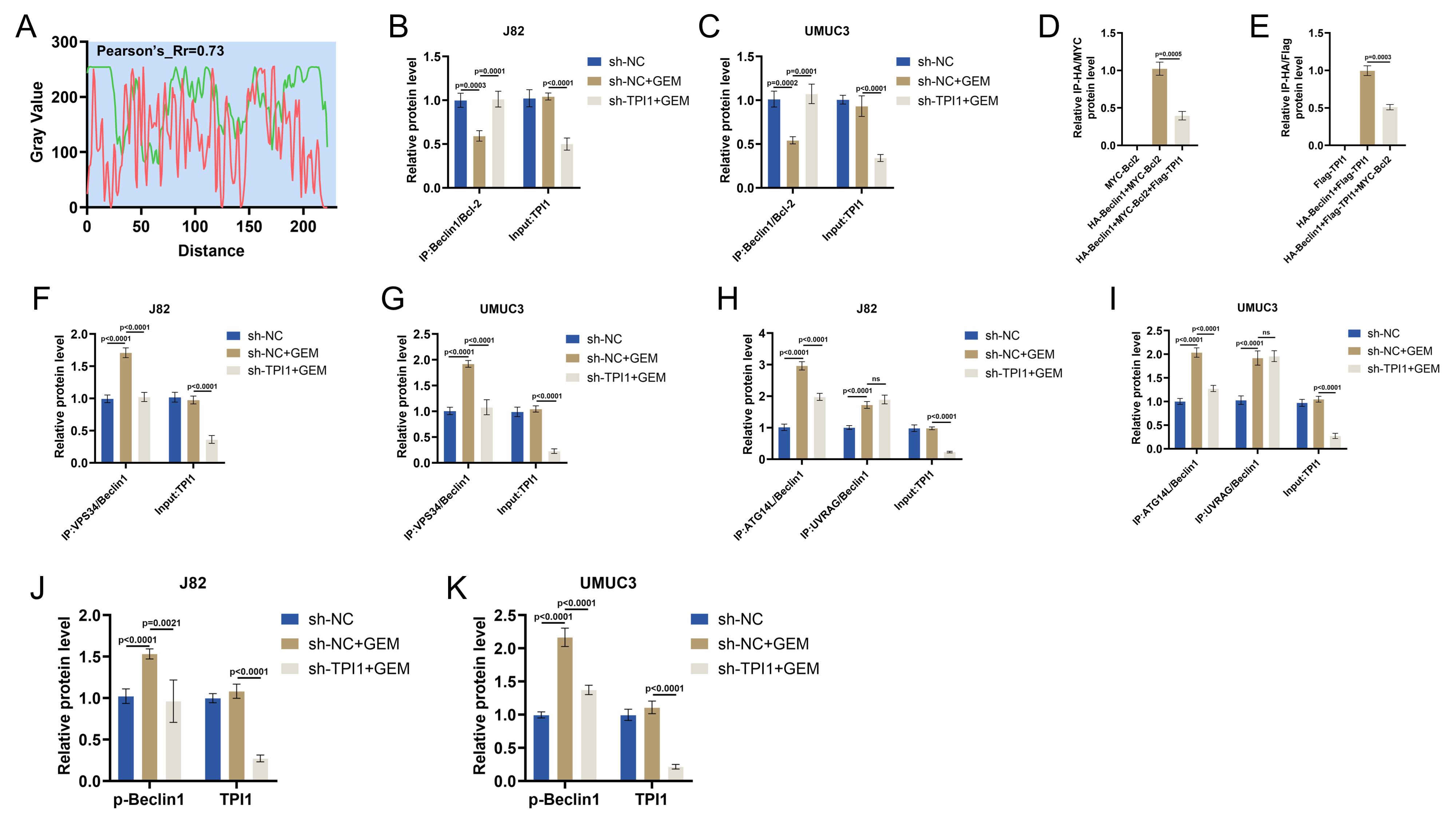

Supplement: Supplementary file 5 — Figure S2 [file 41419_2025_8368_MOESM5_ESM.tif]

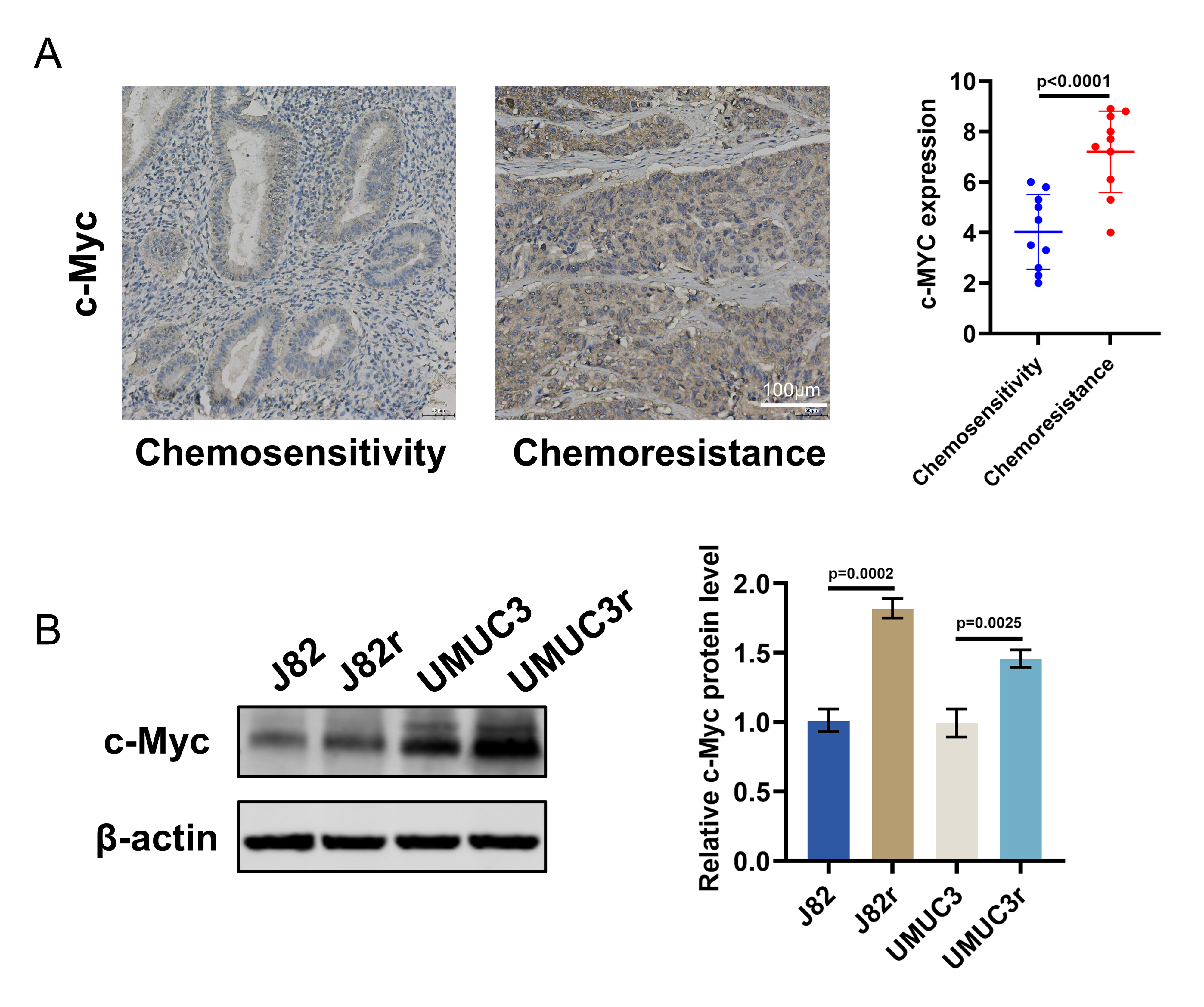

Supplement: Supplementary file 6 — Figure S3 [file 41419_2025_8368_MOESM6_ESM.tif]
